# Progress on the Anti-Inflammatory Activity and Structure–Efficacy Relationship of Polysaccharides from Medical and Edible Homologous Traditional Chinese Medicines

**DOI:** 10.3390/molecules29163852

**Published:** 2024-08-14

**Authors:** Yuanyuan Zhang, Xiulian Lin, Li Xia, Suhui Xiong, Bohou Xia, Jingchen Xie, Yan Lin, Limei Lin, Ping Wu

**Affiliations:** 1 School of Pharmacy, Hunan University of Chinese Medicine, Changsha 410208, China; 20223741@stu.hnucm.edu.cn (Y.Z.); 20232047@stu.hnucm.edu.cn (X.L.); 15376155784@163.com (L.X.); xsh1994@126.com (S.X.); xiabohou@163.com (B.X.); xiejingchen2022@163.com (J.X.); linyan198210@163.com (Y.L.); 2 Key Laboratory for Quality Evaluation of Bulk Herbs of Hunan Province, Hunan University of Chinese Medicine, Changsha 410208, China

**Keywords:** anti-inflammatory, medicinal food, polysaccharide, conformational relationship, Toll-like receptor-related signaling pathway, MAPK signaling pathway

## Abstract

Medicinal food varieties developed according to the theory of medical and edible homologues are effective at preventing and treating chronic diseases and in health care. As of 2022, 110 types of traditional Chinese medicines from the same source of medicine and food have been published by the National Health Commission. Inflammation is the immune system’s first response to injury, infection, and stress. Chronic inflammation is closely related to many diseases such as atherosclerosis and cancer. Therefore, timely intervention for inflammation is the mainstay treatment for other complex diseases. However, some traditional anti-inflammatory drugs on the market are commonly associated with a number of adverse effects, which seriously affect the health and safety of patients. Therefore, the in-depth development of new safe, harmless, and effective anti-inflammatory drugs has become a hot topic of research and an urgent clinical need. Polysaccharides, one of the main active ingredients of medical and edible homologous traditional Chinese medicines (MEHTCMs), have been confirmed by a large number of studies to exert anti-inflammatory effects through multiple targets and are considered potential natural anti-inflammatory drugs. In addition, the structure of medical and edible homologous traditional Chinese medicines’ polysaccharides (MEHTCMPs) may be the key factor determining their anti-inflammatory activity, which makes the underlying the anti-inflammatory effects of polysaccharides and their structure–efficacy relationship hot topics of domestic and international research. However, due to the limitations of the current analytical techniques and tools, the structures have not been fully elucidated and the structure–efficacy relationship is relatively ambiguous, which are some of the difficulties in the process of developing and utilizing MEHTCMPs as novel anti-inflammatory drugs in the future. For this reason, this paper summarizes the potential anti-inflammatory mechanisms of MEHTCMPs, such as the regulation of the Toll-like receptor-related signaling pathway, MAPK signaling pathway, JAK-STAT signaling pathway, NLRP3 signaling pathway, PI3K-AKT signaling pathway, PPAR-γ signaling pathway, Nrf2-HO-1 signaling pathway, and the regulation of intestinal flora, and it systematically analyzes and evaluates the relationships between the anti-inflammatory activity of MEHTCMPs and their structures.

## 1. Introduction

The basic theory of “medicinal and edible homologues” first appeared in the “Yellow Emperor’s Classic of Internal Medicine,” which proposed the concept of the same origin of food and medicine and the concept of the prevention of diseases before they occur and, through dietary therapy, dietary supplements, or medicinal diets, etc., regulating the organism and improving the human body’s immunity to prevent diseases, i.e., preventing diseases before they occur. According to the theory of medicinal and edible homologues, medicinal and edible homologous varieties are effective at preventing and treating chronic diseases and in health care, reflecting the medicinal function of food [1]. With the development of food, medicine, and other fields, the theory of medicinal and edible homologues is also constantly being enriched and improved, including information on ginseng, Astragalus, wolfberry, and other traditional Chinese medicines included in the Catalog of Medicinal and Edible Homologues. In 2012, the National Health and Planning Commission announced a total of 86 types of Chinese herbs in the catalogue “according to the tradition of both food and medicine,” 15 types of new Chinese herbs were added in 2014, and 9 types of new Chinese herbs were added in 2018. As of 2022, the total number of herbs published by the National Health and Planning Commission was 110 [2]. Moreover, because of the rich bioactive substances in MEHTCMs, they have been developed into a series of related functional products as characteristic resources, which fully reflects the combination of traditional Chinese medicine ansd modern concepts.

The inflammatory response, due to the activation of the body’s innate and adaptive immune responses to pathogenic factors, is the body’s first line of defense against harmful stimuli, but excessive and persistent inflammatory responses can seriously affect the health of the body. In contemporary life, work and life stress, unhealthy dietary habits, bacteria and viruses, tissue damage, or necrosis can lead to varying degrees of inflammation, and if not treated in a timely manner, acute inflammation can further shift to chronic inflammation, leading to the occurrence of various diseases [3,4,5,6,7]. Therefore, the control and treatment of inflammation are particularly important and necessary to guard against subsequent disease processes. However, some traditional anti-inflammatory drugs such as nonsteroidal anti-inflammatory drugs (NSAIDs), which are widely used in clinical practice at present, have significant anti-inflammatory activity, but they all have some adverse effects, among which gastrointestinal adverse effects are the most common [8,9,10,11]. In this context, the research and development of new anti-inflammatory drugs has become the focus of clinical research, and an increasing number of researchers have begun to turn this research direction to MEHTCMs. At the same time, many studies on food nutrition and health have shown that some functional components of MEHTCMs can inhibit, alleviate, and improve inflammation without toxic side effects [12,13] and are potential natural anti-inflammatory drugs.

MEHTCMPs are polyhydroxy derivatives containing keto groups or aldehyde groups formed by the polymerization and dehydration of more than 10 monosaccharide molecules of the same or different types through glycoside bonds [14]. Due to their advantages of low toxicity, high safety, extensive functions, and significant antitumor activity [15], antioxidant activity [16], hypoglycemic activity [17], antiviral activity [18], immunomodulatory activity [19], anti-inflammatory activity [20], and other biological activities, the development and research of MEHTCMPs have become a topic of concern in traditional Chinese medicine (TCM) innovation research in recent years. In addition, MEHTCMPs have broad application prospects. Because of the clinical need to urgently develop new anti-inflammatory drugs to make up for the shortcomings of nonsteroidal anti-inflammatory drugs, the anti-inflammatory activity of MEHTCMPs has attracted the attention of many researchers, due to the multiple targets responsible for the anti-inflammatory activity, and the number of reports is increasing [21]. The anti-inflammatory activity of more prominent MEHTCMPs, including *Lycium barbarum* polysaccharides, *Dendrobium* polysaccharides, and *Astragalus membranaceus* polysaccharides, has been increasingly studied. Studies have shown that *Dendrobium huoshanense* polysaccharide (cDHPS) has a protective effect on CIA mice. It can alleviate joint swelling, synovial hyperplasia, ubiquitin formation, cartilage erosion, and bone destruction in CIA mice [22]. *Lycium barbarum* polysaccharides (LBPs), on the other hand, remodel the composition of the intestinal flora, repair intestinal barrier damage, and alleviate liver inflammation in NAFLD rats [23]. In addition, studies have shown that the structures of polysaccharides, such as the molecular weight, monosaccharide composition, and glycosidic bonds, are key for the biological activity of polysaccharides [24]. Therefore, researchers usually use structural modifications to effectively change the structure of polysaccharides and improve their biological activity [25,26]. Based on this information, the study of the structure of MEHTCMPs has become a necessary means to effectively analyze their anti-inflammatory activities. However, because of the unusual complexity of the chemical structures of polysaccharides and the limitations of current technology, the structures of a large number of MEHTCMPs have not been fully elucidated, and the relationship between their structures and anti-inflammatory activity remains unclear, which is a weak point in the research process of MEHTCMPs and a difficult point in the process of developing them as new anti-inflammatory drugs. In view of the importance of these research areas, this paper comprehensively and systematically summarizes the latest progress on the anti-inflammatory molecular mechanism and the relationship between the structure and efficacy of MEHTCMPs. This study provides some theoretical support for the further development and utilization of MEHTCMPs and provides new insights for the development and utilization of new anti-inflammatory drugs.

## 2. Anti-Inflammatory Mechanism of MEHTCMPs

**Figure 1:**
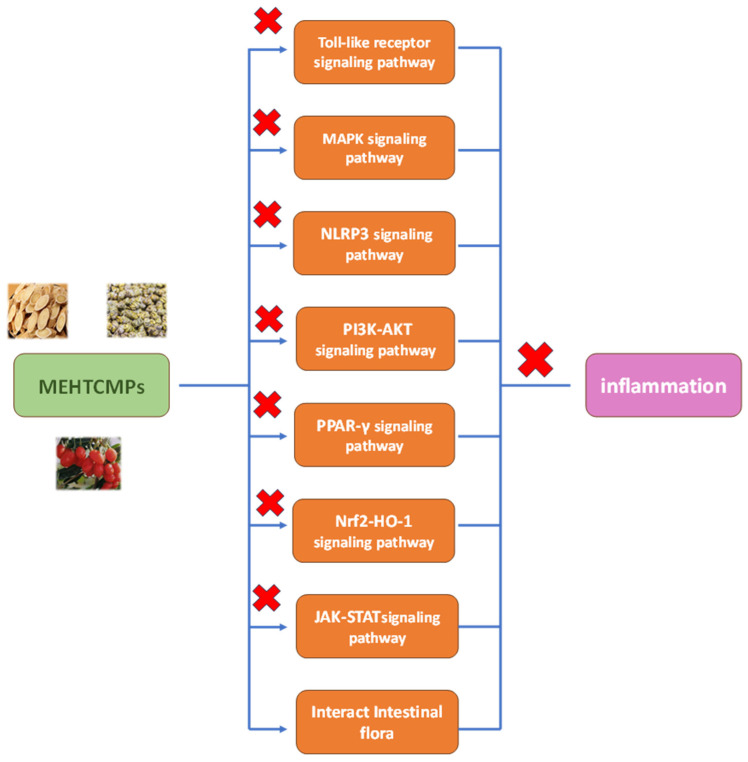
Anti-inflammatory effects of MEHTCMPs through different pathways.

Since inflammatory responses are involved in many complex disease processes, the research and development of anti-inflammatory drugs is of particular importance. The intricate relationship between anti-inflammatory mechanisms and inflammatory diseases highlights the link between the two. Notably, the anti-inflammatory activity of MEHTCMPs has been proven by a large number of related studies; therefore, an in-depth study of the anti-inflammatory mechanism of MEHTCMPs has a very important role in the subsequent development of MEHTCMPs into novel anti-inflammatory drugs. As shown in Figure 1, MEHTCMPs exert anti-inflammatory effects via the Toll-like receptor signaling pathway, MAPK signaling pathway, NLRP3 signaling pathway, PI3K-AKT signaling pathway, PPAR-γ signaling pathway, Nrf2-HO-1 signaling pathway, and JAK-STAT signaling pathway and the regulation of intestinal flora.

### 2.1. Toll-like Receptor Signaling Pathways

**Figure 2:**
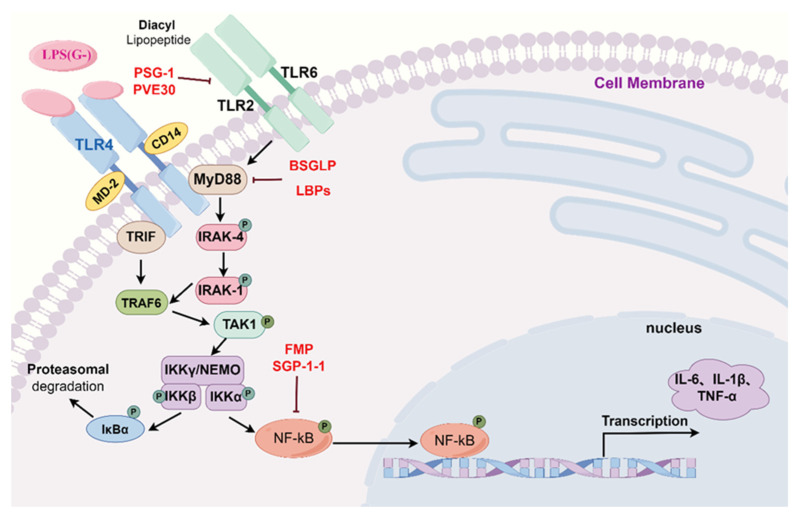
Effects of MEHTCMPs on the signaling pathway of TLRs. ⊥ represents inhibition and → represents promotion. First, LPS binds to LBP to form the LPS–LBP–CD14 complex. Then, TLR4 recognizes the complex LPS–LBP–CD14 via MD-2. This in turn activates the MyD88-dependent pathway, which transduces LPS-stimulated signals downstream, and ultimately activates the NF-κB signaling pathway, which regulates the release of inflammatory factors such as TNF-α and IL-1β. By Figdraw 2.0.

Toll-like receptors (TLRs) recognize different pathogen-associated molecular patterns and play integral roles in the innate immune response. They are the first line of defense against pathogen invasion and play key roles in inflammation, immune cell regulation, survival, and proliferation [27]. Different TLRs recognize different pathogenic microorganisms, and the study of TLR4 and its downstream signaling pathway has received extensive attention because of its important role in the immune response and inflammatory response [28,29,30,31,32,33,34,35]. Studies have shown that MEHTCMPs, such as *Ganoderma lucidum* polysaccharides, *Lycium barbarum* polysaccharides, Fructus mori polysaccharides, and *Siraitia grosvenorii* polysaccharides, exert their anti-inflammatory effects mainly by inhibiting the TLR-MyD88-NF-κB signaling pathway, as shown in Figure 2. *Ganoderma lucidum* polysaccharide and *Lycium barbarum* polysaccharide have therapeutic effects on hepatic inflammation. SANG T et al. reported that 300 mg/kg and 100 mg/kg *Ganoderma lucidum* polysaccharide (BSGLP) alleviated localized inflammation and fat accumulation in the livers of HFD-fed C57BL/6J mice. BSGLP significantly decreased the serum levels of the pro-inflammatory factors TNF-α, IL-1β, IL-6, and MCP-1 and reduced the Firmicutes/Bacteroidetes ratio in mice, while also significantly reducing Myd88 and TLR4 expression in mouse adipose tissue [36]. Aerobic training and treatment with *Lycium barbaru* polysaccharide (LBPs) at a dose of 50 mg/kg reduced IL-6, IL-1β, and TNF-α release from plasma and TLR4, MyD88, p38MAPK, and p-NF-κB p65 expression in the liver tissues of NAFLD rats; downregulated intestinal-derived lipopolysaccharide and hepatic lipopolysaccharide-binding protein expression; and increased ZO-1 and occludin expression, exerting hepatoprotective effects [23]. In addition, *Fructus mori* polysaccharide (FMP) and *Siraitia grosvenorii* polysaccharide (SGP-1-1) can treat inflammation caused by type 1/2 diabetes mellitus. Chen X et al. reported that 600 mg/mL FMP significantly inhibited the expression of TLR4, MyD88, p-IKKβ, and p-NF-κB p65 in the gut of T2DM mice and reduced the serum levels of TNF-α, IL-1β, and IL-6. The expression of claudin-1, occludin, and ZO-1 and the level of IL-10 increased to repair damage to the intestinal barrier and thus relieve intestinal inflammation and oxidative stress [37]. However, 50, 100, and 200 mg/kg SGP-1-1 can significantly inhibit the expression of TLR4 and NF-κB p65 mRNA in the kidneys of DN mice and stimulate the production of SOD, thus reducing the release of IL-6, TNF-α, and MDA and alleviating the damage caused by inflammation and oxidative stress in DN mice [38]. In addition, *Ganoderma lucidum* polysaccharide (PSG-1) and *Codonopsis pilosula* polysaccharide (CPP1-2-1) can alleviate inflammatory damage in mice with colitis. YING M et al. reported that 25, 50, and 100 mg/kg PSG-1 can effectively regulate the mRNA levels of TLR-2, TLR-4, and TLR-6 in Cy-induced colitis mice. Thus, the release of TNF-α, IL-1β, and IL-2 is reduced [39]. CPP1-2-1, on the other hand, reduces the expression of TLR4, NF-κB, TNF-α, and IL-6 in LPS-induced RAW264.7 cells in a dose-dependent manner and alleviates DSS-induced pathological injury in mice with colitis [40]. In addition, ZHAO Y et al. showed that a 600 mg/kg dose of *Glycyrrhiza uralensis* polysaccharide (GCP) significantly reduced the mRNA levels of IL-1β, IL-6, TNF-α, TLR-4, MyD88, and NF-κB; increased the serum levels of IL-4 and IL-10; and effectively alleviated hypothalamic inflammation in AA broilers [41]. LIU T et al. reported that 200 mg/kg *Astragalus membranaceus* polysaccharide (AP) inhibited CVB3-induced VM and that AP significantly reduced the expression of IL-1β, IL-6, TNF-α, INF-γ, MCP-1, TLR-4, and p-NF-κB p65 in CVB3-induced mouse hearts; moreover, the serum CK-MB, AST, LDH, LVEF, and LVFS levels were also significantly reduced after AP treatment [20]. PVE30, a polysaccharide isolated from *Prunella vulgaris* L., is a potential therapeutic drug against HSV. ZHONG X et al. reported that 5, 10, 20, and 40 μg/mL PVE30 significantly inhibited TLR2 expression in HeLa cells, which led to an inhibition of NF-κB activation and a reduction in IL-6 and TNF-α levels [42].

### 2.2. MAPK Signaling Pathway

The mitogen-activated protein kinase (MAPK) signaling pathway mainly consists of p38 MAPK, c-Jun N-terminal kinase (JNK), and extracellular signal-regulated protein kinase (ERK) [43]. When stimulated by external factors, the three proteins are phosphorylated, which in turn activates the expression of the corresponding downstream proteins and regulates the release of inflammatory factors. Studies have shown that the MAPK signaling pathway is involved in the development of many inflammation-related diseases [44,45,46,47,48]. Moreover, MAPKs are often cross-linked with the NF-κB signaling pathway, which together participate in regulating inflammatory responses [49,50,51,52].

#### 2.2.1. P38-NF-κB Signaling Pathway

**Figure 3:**
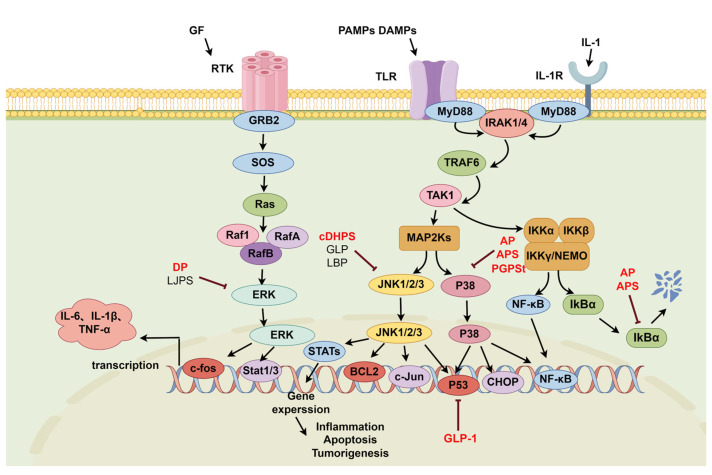
Effects of MEHTCMPs on the MAPK signaling pathway. ⊥ represents inhibition and → represents promotion. First, JNK and P38 are activated by activated TLRs and IL-1R, which interact with the junction protein MyD88, phosphorylating IRAK-1/4 and interacting with TRAF6 to activate TAK1, which in turn activates MKK3/6 and MKK4/7, leading to the activation of JNK and p38 MAPK and the modulation of downstream protein expression, regulating the release of inflammatory factors. ERK is mainly activated by RTK and requires the participation of Ras, PKC, and Raf proteins. Normally, ERK is located in the cytoplasm, but once activated, ERK rapidly crosses the nuclear membrane and activates transcription factors such as STATs and Fos, thus regulating the release of inflammatory factors. By Figdraw2.0.

P38 is a very important MAPK pathway and a relay station for cellular signaling [53] When stimulated by lipopolysaccharide, physiological stress, UV irradiation, and osmotic stress, p38 is activated, enters the nucleus, and acts on corresponding transcription factors to regulate the expression and release of a variety of inflammatory factors, such as IL-1 and COX-2, which are potential targets for the treatment of inflammatory diseases [54]. *Ganoderma lucidum* polysaccharides, *Angelica sinensis* polysaccharides, *Astragalus membranaceus* polysaccharides, and *Platycodon grandiflorus* polysaccharides can play a role in slowing inflammation by inhibiting the activation of the P38-NF-κB signaling pathway, as detailed in Figure 3, leading to the effective treatment of cognitive dysfunction, intestinal inflammation, liver injury, and other diseases. ZHANG Y et al. reported that *Ganoderma lucidum* polysaccharide (GLP-1) could ameliorate cognitive dysfunction in D-gal-induced rats by modulating brain–liver axis inflammation and inflammation-induced metabolic pathway disorders. The results showed that administration of a 20 mg/kg dose of GLP-1 for 60 days significantly reduced blood ammonia levels and the levels of the pro-inflammatory factors TNF-α, IL-6, p-p38 MAPK, and p-p53 and increased the release of IL-10 and TGF-β1 in the liver and brain tissues of D-gal-induced rats [55]. TIAN M et al. found that *Angelica sinensis* polysaccharide (AP) exhibited a good anti-inflammatory activity in LPS-induced claw dermal cells and is a potential drug for the treatment of hoof laminitis [56]; at doses of 10, 50, and 100 µg/mL, AP significantly inhibited the phosphorylation of p38, IκBα, and p65 in LPS-induced claw dermal cells, which resulted in reductions in TNF-α, IL-1β, IL-6, and NO production and the reduced mRNA expression of the pro-inflammatory factors CCL2, CCL20, etc. *Astragalus membranaceus* polysaccharide (APS) is a potential supplement to enhance intestinal immunity, and DONG N et al. found that 200 mg/kg APS significantly suppressed the expression levels of p-p38 MAPK and p-NF-κB p65 and significantly increased the expression of IκB-α protein in LPS-induced IPEC-J2 cells. Moreover, APS also significantly inhibited the expression of IL-6, IL-1β, TNF-α, and chemokines in the jejunal tissues of LPS-induced BALB/c mice and significantly improved the integrity of the jejunal villi in mice [57]. In addition, QI C et al. reported that a 200 mg/kg dose of *Platycodon grandiflorus* polysaccharide (PGPSt) improved the structure of mouse hepatocytes; attenuated hepatocellular injury; significantly reduced AST, ALT, and SOD activities and the levels of IL-6, IL-1β, TNF-α, and MDA in liver tissues; downregulated the expression of cleaved caspase-3 and Bax in liver tissues; upregulated the expression of the Bcl-2 and GSH proteins; and inhibited hepatocyte apoptosis and TLR4, p-P38, and NF-κB p65 protein expression. Thus, it effectively ameliorated LPS/D-GalN-induced acute liver injury in mice [21].

#### 2.2.2. JNK-NF-κB Signaling Pathway

JNK is an important branch of the MAPK pathway that plays an important role in a variety of physiological and pathological processes, such as the cell cycle, reproduction, apoptosis, and cellular stress [58]. Studies have shown that JNK activation is closely associated with chronic inflammation and tumorigenesis [59,60]. MEHTCMPs can effectively inhibit JNK activation and thus alleviate inflammation, as shown in Figure 3.

SHANG Z Z et al. reported that a low dose of 0.1095 g/kg and a high dose of 0.4380 g/kg *Dendrobium huoshanense* polysaccharide (cDHPS) had therapeutic effects on RA. The results showed that cDHPS dose-dependently remodeled Th17 and Treg homeostasis; decreased MMP3 and MMP8 levels in the synovial tissue and serum of CIA mice with type II collagen-induced arthritis; decreased MMP9, IL-1β, IL-6, IL-17, TNF-α, GM-CSF, M-CSF, CXCL12, and CCL5 levels in the synovial tissues and serum of CIA mice with type II collagen-induced arthritis; inhibited HIF-1α expression; and promoted the release of IL-10 and TGF-β1. In addition, cDHPS significantly reduced the phosphorylation of IκB, p65, and JNK in joint tissues, effectively alleviating joint swelling, synovial hyperplasia, and bone destruction in CIA mice [22]. GUO C et al. reported that water-soluble *Ganoderma lucidum* polysaccharide (GLP) at doses of 200 and 300 mg/kg dose-dependently activated GPR43 in mouse colon cells in response to changes in the composition of the intestinal microbiota and an increase in the production of SCFAs; significantly inhibited the expression of TLR4, MyD88, p-NF-κB p65, p-JNK, and p-ERK; and reduced the expression of IL-1β, IL-6, and TNF-α and iNOS, COX-2, and MCP-1 mRNAs in the serum of AOM/DSS-induced mice; alleviated colitis and tumorigenesis; and reduced the size and overall number of tumors in AOM/DSS-induced mice. Moreover, GLP also significantly inhibited the phosphorylation of ERK and JNK and decreased the levels of IL-6, IL-1β, TNF-α, iNOS, and COX-2 in LPS-induced RAW264.7, HT-29, and NCM460 cells [61]. XIAO J et al. reported that *Lycium barbarum* polysaccharide (LBPs) had potential protective effects on liver injury in HFD-induced NAFLD rats, and experiments showed that a 1 mg/kg dose of LBPs significantly reduced fat deposition, inflammation, and ALT levels in rat livers; significantly reduced the levels of iNOS, COX-2, IL-1β, SOCS-3, TGF-β1, and α-smooth muscle actin (a-SMA) mRNAs and TGF-β1 and CYP2E1 protein levels in liver tissue; and inhibited JNK/c-Jun phosphorylation, thus improving liver fibrosis and alleviating oxidative stress-induced liver injury [62].

#### 2.2.3. ERK-NF-κB Signaling Pathway

ERK is an important member of the MAPK family, and once ERK is activated and translocated into the nucleus, it activates transcription factors such as NF-κB and AP-1 [63]. Studies have shown that the abnormal activation of ERK is closely related to the pathological processes of many inflammatory diseases [64,65,66,67]. The polysaccharides of medicinal food and traditional Chinese medicine can prevent the abnormal activation of ERK and block the inflammatory process, as shown in Figure 3.

TIAN Hua et al. reported that a 60 mg/kg dose of *dandelion* polysaccharide (DP) significantly reduced the levels of IL-6, TNF-α, and PGE2 and the expression of the iNOS, COX-2, and ph-ERK1/2 proteins and increased the content of IL-10 in the gastric tissues of rats. These findings suggested that DP may alleviate *H. pylori*-associated gastritis by inhibiting the activation of the MAPK-ERK pathway, thereby reducing the release of pro-inflammatory factors [68]. WANG S et al. reported that sulfated *seaweed* polysaccharide (LJPS) reduced LPS-induced ERK and IKKα/β phosphorylation, as well as PGE2, TNF-α, and IL-1β release, in RAW264.7 cells in a dose-dependent manner, with the most significant inhibitory effect of LJPS observed at a dose of 400 µg/mL, for an inhibition rate of 62.15% [69].

### 2.3. The NLRP3 Signaling Pathway

The Nod-like receptor protein 3 (NLRP3) inflammasome is a fully functional pattern recognition receptor that plays important roles in immune regulation and the development of many inflammatory diseases [70]. Its activation induces the maturation and secretion of the pro-inflammatory factors IL-1β and IL-18, and studies have shown that NLRP3 activation is associated with the pathogenesis of a variety of diseases, including gout, type 2 diabetes mellitus, and Alzheimer’s disease [71,72].

**Figure 4:**
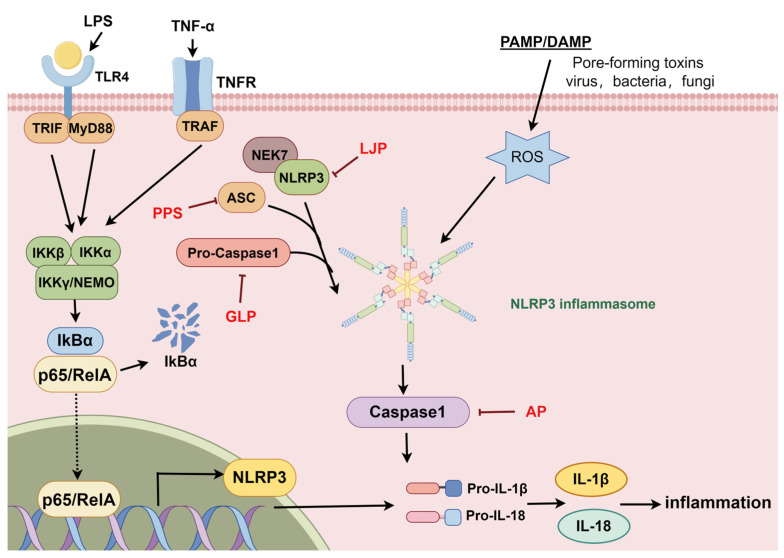
Effects of MEHTCMPs on the NLRP3 signaling pathway. ⊥ stands for inhibition, → stands for promotion. The NLRP3 inflammatory vesicle is activated in 2 steps. First, PAMP- or DAMP-mediated activation of TLR4 or TNFR induces NF-kB signaling, leading to elevated expression of NLRP3, pro-IL-1β, and pro-IL-18 (step 1) Next, a large number of signals such as PAMP/DAMPs indirectly activate NLRP3, leading to complex assembly and caspase-1 activation (step 2). Activated caspase-1 induces secretion of the pro-inflammatory cytokines IL-1β and IL-18, leading to inflammation. By Figdraw2.0.

*Lonicera japonica* polysaccharide, *Poria cocos* polysaccharide, *Polygonatum sibiricum* polysaccharide, *Ganoderma lucidum* polysaccharide, and *Angelica sinensis* polysaccharide can regulate NLRP3 to treat depression, neuroinflammation, liver injury, and chronic renal failure diseases, as shown in Figure 4.

Ping Liu et al. reported that 30 and 100 mg/kg *Lonicera japonica* polysaccharide (LJP) can significantly downregulate the expression of NLRP3, caspase-1, and IL-1β in the hippocampus of a mouse model of chronic unpredictable mild stress (CUMS). Thus, it has a protective effect on depressed mice [73]. However, Shi et al. reported that 20 and 80 mg/kg *Poria cocos* polysaccharide (PPS) significantly reduced the levels of IL-1β, IL-18, and TNF-α in the hippocampus of mice with LPS-induced depression and downregulated the expression of CD16/32, NF-κB p65, NLRP3, ASC, and cleaved caspase-1. Moreover, CD206 expression was upregulated, thus alleviating the anxiety and depression-like behavior induced by LPS in mice. In addition, 4, 8, and 16 μmol/L PSS inhibited the LPS-induced polarization of BV-2M1 cells and significantly reduced the levels of the inflammatory factors ROS, NO, TNF-α, and IL-1β in BV-2 cells. Moreover, it promoted the polarization of M1 microglia to the M2 phenotype by regulating CD16/32 and CD206 [74]. Additionally, Han Li et al. reported that 50 μg/mL *Ganoderma lucidum* polysaccharide (GLP) significantly inhibited the expression of NF-κB, NLRP3, ASC, pro-caspase-1, caspase-1, IL-1β, TNFα, and IL-17 while upregulating the expression of Dectin-1 and IL-10 in an LPS-induced microglial inflammation model of BV2 cells, and 5 mpk doses of GLP produced the same effect as described above in both a 0.2% cuprizone-induced CNS demyelinating disease mouse model and a MOG35-55-induced EAE inflammatory demyelinating mouse model, suggesting that GLP may alleviate neuroinflammation and ameliorate neuroinflammation by modulating the Dectin-1 receptor and inhibiting the activation of NF-κB/NLRP3 inflammasome signaling to improve motor function and promote myelin regeneration [75]. XIAO L et al. reported that 150, 300, and 600 mg/kg *Polygonatum sibiricum* polysaccharide (PSP) can reduce 48 h mortality and attenuate histopathological damage to the liver in LPS-induced septic acute liver injury (SALI) mice. PSP reduced the levels of the hepatic function indices AST, ALT, ALP, and TBIL and the levels of MPO, TNF-α, and IL-6 in liver tissues, as well as the serum levels of the pro-inflammatory factors IL-18 and IL-1β, which are associated with cellular pyrolysis. Furthermore, PSP significantly inhibited the expression of GSDMD-NT and reversed the increase in the mRNA expression levels of NLRP3/GSDMD signaling components in liver tissues [76], which has a protective effect on acute liver injury in sepsis. Wan Hongbo et al. reported that treatment with *Angelica sinensis* polysaccharide (AP) at doses of 100, 200, and 400 mg/kg could alleviate pathological damage to renal tissue and improve renal function in CRF rats in a dose-dependent manner. It significantly reduced the levels of Scr, BUN, and 24 h urinary protein in CRF rats, as well as the levels of MDA, NLRP3, caspase-1, and IL-1β in renal tissue and the levels of IL-18, IL-1β, and IL-6 in the serum [77].

### 2.4. The PI3K-AKT Signaling Pathway

The phosphatidylinositol 3-kinase/protein kinase B (PI3K-Akt) signaling pathway is an intracellular signaling pathway that responds to extracellular signals to promote metabolism, proliferation, cell survival, growth, and angiogenesis. When PI3K is activated, Akt is phosphorylated, which in turn activates downstream proteins such as NF-κB, mTOR, and GSK3β, thereby regulating physiological processes such as cell proliferation, cell death, and inflammatory responses [78,79,80].

#### 2.4.1. PI3K-AKT-GSK3β

GSK3β is a downstream target protein of the PI3K-AKT pathway and a key kinase in the inflammatory response. GSK-3β dysfunction is closely associated with inflammatory diseases such as heart failure and cancer [81,82]. Therefore, studying the functional activity of GSK-3β and its inhibitors has become a potential target for the treatment of numerous diseases.

Huang Hong et al. reported that *Codonopsis pilosula* polysaccharide (CPP) could alleviate herpes simplex virus encephalitis (HSE), and 100 mg/kg CPP significantly increased the expression of p-AKT, AKT, p-GSK3β, and GSK3β in the brain tissues of HSV-I mice and decreased the serum levels of IL-1β, NO, MDA, ROS, IFN-γ, and S-100B, as well as the viral titers in the brain, liver, and lung tissues. Further experiments showed that the administration of the PI3K-AKT/GSK3β inhibitor LY294002 in combination with CPP attenuated the protective effect of CPP on the brain tissues of HSV-I mice, suggesting that the PI3K-AKT-GSK3β pathway is an important signaling pathway for CPP in the treatment of type I HSE [83].

#### 2.4.2. PI3K-AKT-mTOR

mTOR is an important regulator of cell growth and proliferation and plays a major role in the regulation of cellular bioactivity, protein translation, and inflammatory responses [84,85].

Low-molecular-weight *ginseng* polysaccharide (LGP) and *Polygonatum sibiricum* polysaccharide (PSP) improved ConA-induced autoimmune hepatitis and CCl4-induced acute liver injury, respectively. QI X et al. reported that LGP doses of 200 mg/kg and 400 μg/mL could effectively reduce ConA-induced hepatitis in C57BL/6 mice and RAW264.7 cells. The levels of AST, ALT, TNF-α, IL-18, IL-6, IL-1β, p-PI3K, p-AKT, p-mTOR, and p-TAK1 in cells are used to treat autoimmune hepatitis [86]. Zhang Xinxin’s research showed that 400 mg/kg PSP could effectively improve the dull yellow and rough surface of CCL4-induced liver tissue in rats, significantly reduce the number of necrotic and inflammatory cells and the expression of p62, and inhibit the expression of p-PI3K, PI3K, p-AKT, AKT, p-mTOR, and mTOR while increasing expression of the LC3II/LC3I protein [87].

### 2.5. PPAR Signaling Pathway

PPARs are a class of nuclear receptor transcription factor superfamily proteins that mainly consist of three isoforms, PPAR-α, PPAR-β/δ, and PPAR-γ, with PPAR-γ being the most classical; PPARs have been shown to play an important role in the regulation of inflammatory responses [88,89].

XU T et al. reported that 300 μg/mL *Lycium barbarum* polysaccharide (LBP) significantly downregulated COX-2, NLRP3, TNF-α, IL-1β, and IL-6 mRNA and protein expression in LPS-induced bMECs. Phosphorylation of IκBα, p65, p38, JNK, and ERK decreased in a PPARγ-dependent manner [90]. These findings suggest that LBP is a potential agent for preventing and treating mastitis. In addition, Wang Hui et al. reported that a 200 mg/kg dose of *Hippophae rhamnoides* polysaccharide (HRP) could effectively alleviate pathological injury to the liver in septic mice and significantly decrease the expression of NF-κB, Bax, and cleaved caspase-3 and the rate of apoptosis in the liver while increasing the expression of PPARγ and Bcl-2. In addition, in PPARγ knockout septic mice, the expression of NF-κB, Bax, and cleaved caspase-3 increased, while the apoptosis rate and Bcl-2 expression decreased in the livers of the mice, suggesting that PPARγ is involved in regulating the inflammatory response and apoptosis in sepsis-induced liver injury. The subsequent administration of HRP showed that although hepatic injury was ameliorated and the serum ALT and AST levels were also reduced, the aforementioned attenuation of liver injury was significantly weaker than the protective effect of HRP on septic mice expressing PPARγ, which suggests that HRP exerts a protective effect on sepsis-induced liver injury through the upregulation of PPARγ expression [91]. Zhang Huazhi et al. reported that 50, 100, and 200 mg/kg *Hedysarum polybotrys* polysaccharide (HPS) could enhance the SOD and GSH-PX activities in the myocardial tissue of db/db mice with type 2 diabetes in a dose-dependent manner and upregulate the mRNA and protein expression of PPARγ, GLUT-4, and MMP2. In addition, MDA, IL-6, and TNF-α levels and NF-κB, IKKβ, and MMP9 mRNA and protein expression were decreased, which significantly alleviated myocardial tissue inflammation and improved myocardial oxidative stress [92]. In addition, Hu et al. reported that a 500 mg/kg dose of *Polygonatum sibiricum* polysaccharide (PLP) significantly inhibited the expression of the inflammatory factors IL-6, TNF-α, and IL-1β in the colonic tissues of ICR mice and suppressed the transcriptional activities of the downstream pathways, NF-κB and AP-1, by modulating the MAPK and PPAR signaling pathways, alleviating inflammation in LPS-induced RAW264.7 macrophages [93].

### 2.6. Nrf2-HO-1 Signaling Pathway

Nuclear faction erythroid2-related factor 2 (Nrf2) is an important transcriptional regulator in vivo that controls the expression of multiple anti-inflammatory and antioxidant genes and plays a key role in the injury response of organisms [94,95]. Heme oxygenase-1 (HO-1) is a rate-limiting enzyme in the catabolism of heme and an important mediator of the anti-inflammatory and antioxidant effects of Nrf2 [96,97]. Nrf2 and HO-1 are involved in inflammation-related pathological processes in various tissues and organs [98,99].

*Dendrobium officinale* polysaccharide can prevent liver lesions by regulating the Nrf2-HO-1 signaling pathway and alleviating inflammation. CHU W et al. administered D-Gal-induced senescent mice *Dendrobium officinale* polysaccharide (M-DOP), which was ultrasonically treated at a medium power of 50 W/cm2, at doses of 250, 500, and 1,000 mg/kg, and the results of the experiments showed that M-DOP could significantly reduce the release of IL-6, IL-1β, and NO; increase the activities of SOD, CAT, and GSH-Px; and upregulate the expression of the Nrf2, HO-1, and NQO1 mRNAs in the livers of senescent mice, thus effectively alleviating liver injury [100]. In contrast, LIANG J et al. reported that *Dendrobium officinale* polysaccharide (DOPS), with a molecular weight of 393.8 kDa, dose-dependently inhibited the increases in ALT, AST, TG, and TC levels in the serum; reduced the infiltration of CD68+ macrophages and the increases in IL-1β, TNF-α, and MDA levels; increased the SOD and GSH-Px activities; and increased the levels of the Nrf-2, HO-1, and NQO-1 mRNAs and proteins in liver tissues, thus effectively alleviating the secondary inflammation caused by DSS-induced colitis. GSH-Px activity and Nrf-2, HO-1, and NQO-1 mRNA and protein levels were upregulated in liver tissues, thereby effectively alleviating DSS-induced liver injury secondary to colitis [101]. In addition, in another study, a 140 mg/kg dose of *Dendrobium officinale* polysaccharide (DOPS) increased SOD levels in ovariectomized (OVX) mice and mice with D-gal-induced learning and memory impairments; upregulated the expression of Nrf2 and HO-1 in the CA1 and CA3 regions of the mouse hippocampus; decreased the release of MDA, TNF-α, and IL-1β; and inhibited the activation of astrocytes and microglia, which resulted in a significant amelioration of learning and memory impairments and the alleviation of neuroinflammation [102]. Liu Ruonan et al. reported that 5, 10, and 50 mg/L *Poria cocos* polysaccharide (PCP) effectively reduced IL-6, TNF-α, MDA, and LDH release; increased SOD activity; and increased Nrf2, HO-1, and NQO1 expression in MAP-induced RTECs, suggesting that PCP is a potential drug for preventing and controlling urinary stones in goats [103]. XIE P et al. reported that a 400 mg/kg *Polygonatum sibiricum* polysaccharide (PSP) significantly upregulated the expression of Nrf2, HO-1, and GluA1 in mice exposed to single prolonged stress (SPS), thereby preventing SPS-induced PTSD-like behavior and synaptic damage [104]. Li H N et al. showed that 8 weeks of continuous administration of a 400 mg/kg dose of *Ganoderma lucidum* polysaccharide (GDLP) significantly increased the expression of the Nrf2 and HO-1 proteins and increased the levels of SOD, CAT, and GSH-Px but significantly decreased the levels of MDA and TNF-α in HFD-fed db/db mice, which had protective effects on T2DM-induced hepatic steatosis, oxidative stress, and inflammation [105].

### 2.7. JAK-STAT Signaling Pathway

The Janus activated kinase/signal transducer and activator of transcription (JAK/STAT) signaling pathway, also known as the IL-6 signaling pathway, is a cytokine-stimulated signaling pathway that was recently identified. This signaling pathway is associated with a variety of functions in organisms and is involved in cell proliferation, differentiation, migration, and apoptosis [106]. Studies have shown that sustained activation of the JAK/STAT signaling pathway is closely associated with many immune and inflammatory diseases [107,108,109,110].

*Angelica sinensis* polysaccharide, *Astragalus membranaceus* polysaccharide, *Dendrobium officinale* polysaccharide, and *Dioscorea polystachya* polysaccharide could exert anti-inflammatory effects by regulating JAK-STAT signaling.

ZHOU Y et al. reported that 80 μg/mL *Angelica sinensis* polysaccharide (AP) significantly enhanced miR-10a expression in LPS-induced HT22 cells and decreased the levels of IL-1β, TNF-α, IL-6, IκBa, p-p65, p-JAK2, and p-STAT3, thereby effectively alleviating epilepsy (EP) [111]. Moreover, WANG K et al. reported that compared with pretreatment, a 6 mg/kg dose of *Angelica sinensis* polysaccharide (ASP) significantly reduced ALT and AST levels, as well as the levels of TNF-α, IFN-γ, IL-2, IL-6, MDA, and ROS, in the liver of ConA-induced mice and increased SOD activity to alleviate oxidative stress; at the same time, ASP inhibited the phosphorylation of proteins related to IL-6/STAT3 signaling and the phosphorylation of NF-κB signaling pathway-related proteins to reduce hepatic inflammatory injury and alleviate liver failure [112]. Wu Tingguo et al. found that 25, 50, and 100 μg/g doses of *Dendrobium officinale* (DOP) dose-dependently and significantly inhibited the expression of p-JAK/JAK and p-STAT3/STAT3 and reduced the levels of IFN-γ, COX-2, and IL-6 in the brain tissues of ICS rats [113]. Sun Yong et al. successfully isolated a *Dioscorea polystachy* polysaccharide (RDPS-I), and observed that 1.0, 2.0, and 3.0 g/kg RDPS-I could effectively regulate the degree of myocardial tissue disarrangement in a dose-dependent manner and the degree of inflammatory cell infiltration; significantly reduce the levels of TNF-α, IL-6, IL-1β, and NF-κB; decrease myocardial tissue expression of p-JAK2/JAK2 and p-STAT3/STAT3; and ameliorate myocardial injury and dysfunction in septic rats [114]. Both *Taraxacum mongolicum* polysaccharide (DP) and *Portulaca oleracea* polysaccharide (POP) had therapeutic effects on TNBS-induced ulcerative colitis. Wang Qian et al. reported that 10 mg/kg DP significantly reduced IL-6 levels and downregulated IL-6Rα and gp130 protein expression and STAT3 and IL-6 mRNA transcript levels in rat colon tissues, thereby alleviating inflammation in colon tissues and protecting and repairing mucosal tissues [115]. Similarly, Fan et al. reported that a 10 mg/kg dose of POP also significantly slowed intestinal mucosal edema and reduced the serum levels of IL-6, IL-6Rα, and gp130, as well as the levels of MPO and NF-κB in intestinal tissues in rats [116].

### 2.8. Regulation of the Intestinal Flora

**Figure 5:**
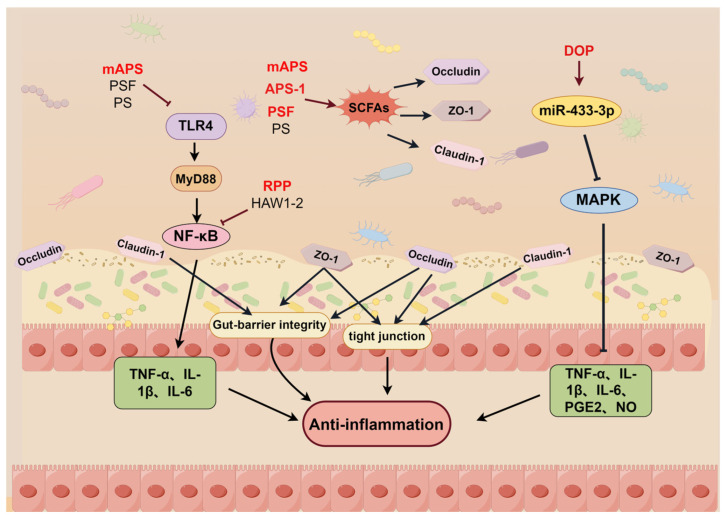
Effects of MEHTCMPs on intestinal flora. ⊥ represents inhibition and → represents promotion. Pharmacophore-derived herbal polysaccharides inhibited the activation of the TLR4/MyD88/NF-κB signaling pathway by increasing the number of intestinal probiotics and inhibiting the growth of harmful flora, in order to reduce the secretion of inflammatory factors in the intestinal tract. SCFAs are one of the important sources of energy in the body, and they can promote intestinal motility and regulate the intestinal pH value, etc. Pharmacophore-derived herbal polysaccharides inhibited the activation of the TLR4/MyD88/NF-κB signaling pathway by increasing the production of SCFAs, thus increasing the expression of ZO-1, occludin, and claudin-1 to repair the damaged intestinal barrier and maintain the physical barrier between cells to exert anti-inflammatory activity. In addition, the polysaccharides of medicinal herbs can increase the expression of miR-433-3p and indirectly inhibit the activation of MAPK, thus suppressing the release of inflammatory factors. By Figdraw2.0.

The intestinal flora is a very large system that plays an indispensable role in human health, and dysregulation of the intestinal flora is closely related to the development of many diseases [117]. Therefore, the relationship between the intestinal flora and human health and disease is one of the key issues of concern at the international academic frontier, and current studies have reported that the intestinal flora plays a positive role in interfering with the development of inflammatory diseases; therefore, the gut may be one of the potential new targets to effectively alleviate the level of inflammation. In recent years, a large number of studies have also reported that herbal extracts can improve the composition and abundance of intestinal flora and repair the intestinal barrier, thereby exerting anti-inflammatory effects by remodeling the composition of the intestinal flora, promoting the growth of probiotics and inhibiting the growth of harmful flora [118,119,120]. However, due to the complexity of the intestinal tract and intestinal flora, its anti-inflammatory mechanism needs to be further explored in the future. MEHTCMPs can exert anti-inflammatory effects by regulating the intestinal flora and repairing the intestinal barrier, as shown in Figure 5.

ZHONG M et al. reported that a 200 mg/kg dose of *Astragalus membranaceus* (mAPS) ameliorated hepatic inflammation and lipid accumulation, reduced HFD-induced body weight gain and elevated ALT and AST levels, decreased the expression of TLR4, NF-κB, and NLRP3, and decreased the Firmicutes/Bacteroidetes ratio in the colon and liver of rats, as well as GPR41 and GPR43 expression, while increasing the abundance of Proteobacteria and Epsilonbacteria and upregulating ZO-1 and OCLN expression to remodel gut microbes [121]. *Pueraria montana var. thomsonii* polysaccharide (RPP), with a molecular weight of 109 KDa, effectively alleviated alcohol- and HFD-induced hepatic injury and steatosis in mice at doses of 50 and 100 mg/kg, respectively, and the results showed that RPP downregulated the expression of TNF-α and inhibited the activation of the NF-κB signaling pathway but upregulated the expression of IL-10, which in turn improved the integrity of the intestinal barrier and regulated the intestinal microbiota composition [122]. In addition, Yang et al. found that continuous oral administration of 200 mg/kg *Astragalus membranaceus* polysaccharide (APS-1) for 8 weeks was an effective treatment for T1D, and the results showed that APS-1 significantly upregulated the expression of ZO-1, occludin, and claudin-1 to improve intestinal barrier function and increased the relative abundance of Muribaculum, Lactobacillus, and Faecalibaculum in the STZ-induced T1D to rebuild the intestinal microbiota, In addition, APS-1 significantly inhibited the expression of IL-6 and TNF-α and increased the release of IL-10 in pancreatic tissues of mice to decrease the level of inflammation, thereby alleviating T1D [123]. In addition, both *Crataegus pinnatifida* polysaccharide (HAW1-2) and *Dendrobium officinale* polysaccharide (DOP) were effective at alleviating intestinal inflammation in mice with DSS-induced colitis. GUO C et al. reported that a 30 mg/kg dose of HAW1-2 significantly inhibited the expression of the inflammatory factors IL-1β, IL-6, and TNF-α, as well as the phosphorylation of IKKα/β, IκBα, and NF-κB, in the colonic tissues of mice. In addition, Alistipes and Odoribacter were significantly enriched, and the production of SCFAs was significantly increased, suggesting that HAW1-2 alleviates intestinal inflammation by remodeling the composition of the intestinal microbiota [124]. Li H et al. reported that 0.5 mg/mL and 200 mg/kg DOP can interfere with the IEC secretion of small extracellular vesicles (DIEs) and regulate the load of miR-433-3p in intestinal sEVs via hnRNPA2B1. Increased expression of miR-433-3p in DIEs is thought to be an important protective factor against intestinal inflammation. DIEs deliver miR-433-3p to LPS-induced macrophages and inhibit the activation of the MAPK signaling pathway by targeting the MAPK8 gene. Thus, the levels of NO, TNF-α, IL-6, and PGE2 in the colon tissue of LPS-induced Caco-2/RAW264.7 cells and DSS-induced colitis mice were decreased [125]. In addition, GU W et al. reported that 120, 240, and 480 mg/kg of *Polygonatum kingianum* polysaccharide (PS) and a high-molecular-weight fraction (PSF) (>100 kDa) dose-dependently alleviated gastrointestinal inflammation and the dysregulation of glucose and lipid metabolism, increased the relative abundance and subsequent SCFA production of SCFA-producing bacteria, and significantly upregulated ZO-1 and occludin expression. In addition, it significantly inhibited TLR4 and IκB-α expression and TNF-α and IL-1β release in liver tissue [126].

The above studies fully suggest that MEHTCMPs can exert anti-inflammatory effects through multiple pathways, among which the anti-inflammatory effects of *Astragalus membranaceus* polysaccharide, *Ganoderma lucidum* polysaccharide, Lycium barbarum polysaccharide, *Dendrobium officinale* polysaccharide, *Dendrobium huoshanense* polysaccharide, *Polygonatum sibiricum* polysaccharide, *Phellinus igniarius* polysaccharide, and *Poria cocos* polysaccharide, which are potential natural anti-inflammatory supplements, are the most prominent and should be investigated in depth in future research on the anti-inflammatory activities of polysaccharides. However, due to the limitations of the current analytical techniques, the structures of MEHTCMPs cannot be fully characterized, and thus the structure–efficacy relationship should be fully studied. Table 1 summarizes 110 kinds of medical and edible homologous traditional Chinese medicines. Table 2 summarizes the anti-inflammatory effects of different MEHTCMPs on different cell/animal models.

## 3. Relationship between the Structures and Anti-Inflammatory Activities of MEHTCMPs

**Figure 6:**
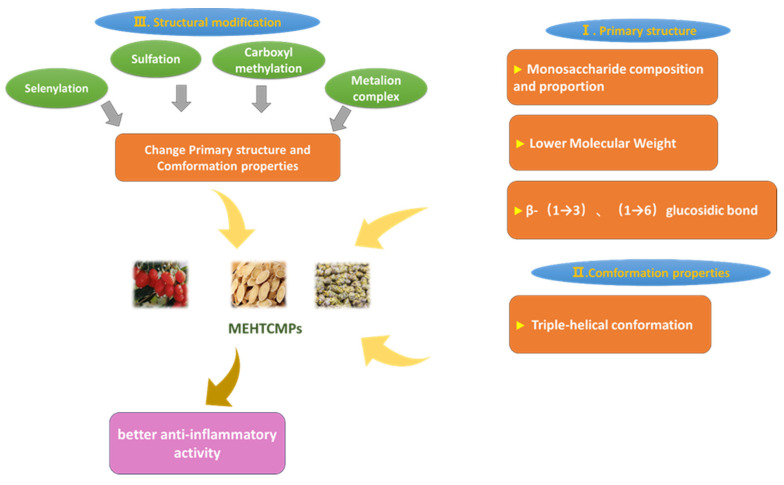
Structure–efficacy relationship of MEHTCMPs.

To a large extent, polysaccharide bioactivity is closely related to polysaccharide structure [149,150,151]. As purely natural polymeric carbohydrates with complex molecular structures, MEHTCMPs are usually categorized into primary structures and advanced structures when structural studies are performed, with the primary structure referring to the planar structure of polysaccharides and the advanced structure referring to the spatial stereo conformation of polysaccharides. Therefore, structural modification is also often used in most studies to change the structure of polysaccharides and achieve enhanced biological activity [152,153,154]. By comparing and summarizing the structures of MEHTCMPs with anti-inflammatory activity, the structure–bioactivity relationships can be deduced, which can provide a theoretical basis for further research on the anti-inflammatory activity of MEHTCMPs. The relationships between the structures of MEHTCMPs and their anti-inflammatory activities are shown in Figure 6.

### 3.1. Primary Structure

At present, most studies on the relationship between the polysaccharide structure and efficacy have focused on the primary polysaccharide structure. The primary structures of polysaccharides are closely related to their biological activities [155,156]. Therefore, current research on the relationship between the activity and structure of MEHTCMPs is mainly based on their primary structures. At the same time, a number of studies have shown that a lower molecular weight, different compositions and proportions of monosaccharides, and β-(1→3), (1→6) glucoside linkages are the main factors affecting the anti-inflammatory activity of METCMPs.

#### 3.1.1. Molecular Weight

The relative molecular mass is an important feature of the structure–efficacy relationship of polysaccharides. A suitable relative molecular mass is the primary condition for the pharmacological activity of MEHTCMPs [157,158,159]. Studies have shown that a lower relative molecular mass enables MEHTCMPs to better exert their anti-inflammatory effects. Zhang X et al. reported that among the nine molecular weight fragments isolated from *Lycium barbarum* polysaccharide (LBP), the LBP fragment with a molecular weight of 34.6 kDa significantly inhibited LPS-induced NO release from RAW264.7 cells [138]. In addition, ZOU Y F et al. isolated four polysaccharides, ASP-H-AP, ASP-B-AP, ASP-T-AP, and ASP-Hb-AP, from the head, body, tail, and whole plant of *Angelica sinensis*, respectively, of which ASP-Hb, with the smallest molecular weight of 67.9 kDa, most significantly inhibited LPS-induced IL-6, IL-1β, TNF-α, and TLR4 expression [160]. GAN and Q et al. isolated two types of polysaccharides, namely PCP and HPCP, from *Polygonatum sibiricum Delar. ex Redoute* and the honey of *Polygonatum sibiricum Delar. ex Redoute*, respectively. HPCP, which has a relatively low molecular weight (5 521 kDa), significantly reduced the levels of the p-IKKβ, p-IκBα, and p-p65 proteins and the IL-1β, TNF-α, and IL-6 mRNAs in mice with LPS-induced acute lung injury (ALI). Moreover, it significantly increased the expression of the p-AMPK and Nrf2 proteins and the HO-1 and NQO-1 mRNAs [141]. Wang Jinhu et al. reported that *Astragalus membranaceus* polysaccharide (APSI-C), which has a molecular weight of 4.5 kDa, can more significantly inhibit the release of NO, TNF-α, and IL-10 from LPS-induced RAW264.7 cells than can APSI-A or APSI-B, which have larger molecular weights [129].

However, some studies reported the opposite results. In previous studies, *Dendrobium huoshanense* polysaccharides (DHP-1) and (DHP-2) with molecular weights of 521.37 and 262.50 kDa inhibited abnormal LPS-stimulated secretion of NO and IL-1β from RAW264.7 cells, but the inhibitory effect of the former was greater than that of the latter [137]. This result indicates that the relationship between the molecular weights of polysaccharides and their biological activities should be fully studied. A summary is given in Table 3.

#### 3.1.2. Composition and Proportion of Monosaccharides

The composition of monosaccharides mainly includes the types and proportions of monosaccharides. The monosaccharides of MEHTCMPs have various compositions, and the compositions of these different monosaccharides determine their biological activities to a certain extent [161,162]. Therefore, the monosaccharide composition of MEHTCMPs has been analyzed and studied. Further study of the anti-inflammatory activity of MEHTCMPs is highly important.

Studies have shown that two *Dioscorea polystachya* polysaccharides (CYP-1) and (YP-1) have a good biological activity. However, LIP et al. reported that CYP-1 has a good anti-inflammatory activity and can inhibit the excessive release of TNF-α and IL-1β from LPS-induced RAW264.7 cells and DSS-induced colitis mice. However, ZHAO et al. found that this enhances immunity. Through further comparison, they found that CYP-1 contained more ribose, rhamnose, arabinose, and xylose than YP-1. This result suggests that these monosaccharides may be important for *Dioscorea polystachya* polysaccharides to exert their anti-inflammatory effects [163,164].

Interestingly, even when the composition of the monosaccharides is similar, the anti-inflammatory activity is also different due to the different molar ratios of each monosaccharide, for example, two *Rubusidaeus* polysaccharides, (L-Ps-1) and (F-Ps-3), TNF-α, iNOS, and IL-6 mRNA expression. However, compared with F-Ps-3, L-Ps-1 had a more significant inhibitory effect, which may be related to the decreased levels of rhamnose and arabinose in the monosaccharide composition of L-Ps-1 and the increase in xylose levels [165]. Similarly, *Astragalus membranaceus* polysaccharides (APS-I) and (APS-II) can significantly decrease the levels of NO and TNF-α and increase the release of IL-10 from LPS-induced mononuclear RAW264.7 macrophages. However, APS-I can better inhibit the release of NO and TNF-α. This property may be related to the presence of more mannose residues in APS-I than in APS-II [127]. In addition, Liu Lina et al. reported that *Phellinus igniarius* polysaccharide (SHP-2-1) can better inhibit the release of NO and IL-1β from LPS-induced RAW264.7 cells than can SHP-1-1, possibly because fucose, galactose, and xylose are less abundant in the monosaccharide composition of SHP-2-1 than in the monosaccharide composition of SHP-1-1, but more glucose is present [145]. Kang et al. obtained four polysaccharides containing 48%, 65%, 69%, and 82% fucose by the step-by-step purification of *Sargassum pallidum* polysaccharide (PPS), which could significantly inhibit NO secretion from RAW264.7 cells exposed to LPS, and the inhibitory effect increased with increasing fucose content [166]. These results indicated that the fucose content may play an important role in the anti-inflammatory activity of Sargassum pallidum polysaccharide. In addition, CHEN H et al. reported that two *Dendrobium nobile* polysaccharides, DNP1 and DNP2, could regulate the release of NO, TNF-α, IL-1β, IL-6, and IL-10 from LPS-induced RAW264.7 cells, and no significant difference in their inhibitory activities was observed. Subsequent analysis of the two polysaccharides revealed that both were composed of mannose and glucose. No significant difference in the molar ratio was detected [136]. A summary is given in Table 4.

The above studies have shown that the composition and proportion of monosaccharides are closely related to the anti-inflammatory activity of MEHTCMPs, which can be summarized as an increase or decrease in the content of one or several monosaccharides, and the existence or disappearance of monosaccharides indirectly affects the biological activity of polysaccharides. However, polysaccharides with the same composition and proportion of monosaccharides showed no difference in biological activity.

#### 3.1.3. Glycosidic Bonds

The type and position of glycosidic bonds also play very important roles in the bioactivity of polysaccharides, and studies have shown that β-(1→3), (1→6) glycosidic bonds play an important role in the bioactivity of polysaccharides, such as hypoglycemia [167], tumor inhibition [168], and the enhancement of immunity [169]. Therefore, β-(1→3), (1→6) glucoside linkages are also very likely to be important structures for the anti-inflammatory activity of MEHTCMPs. 

Li Q et al. reported that *Pueraria montana var. thomsonii* polysaccharide (RPP-2), which has an α-D-1,3-glucan structure, can slow the release of TNF-α in HFD-induced NAFLD mice, reshape the Th17/Treg balance, and potentially treat nonalcoholic fatty liver disease [170]. WANG D et al. reported that *Hericium erinaceus* polysaccharide (EP-1), which has a β-d-Glc(1→3) structure, can significantly increase SOD activity, reduce ROS content, and alleviate oxidative damage, thus exerting anti-gag and anti-UC effects [171,172]. In addition, *Phellinus igniarius* polysaccharide (SHPS-1), which has a 1,3-β-D-GLCP residue structure, can significantly reduce the phosphorylation of STAT-1 and the expression of STAT-1 target genes in LPS-stimulated RAW264.7 macrophages, as well as the release of iNOS and TNF-α, and can reduce the levels of inflammatory factors in mice with enteritis. These effects prevent the occurrence of inflammatory enteritis [144]. Zhao Tian-yu reported that a Phellinus igniarius polysaccharide (A3) with an α-1, 6-D-GALp structure could significantly reduce IL-6, IL-1β, and TNF-α mRNA expression in RAW264.7 cells and mice with LPS-induced ulcerative colitis. It also downregulated the expression of p-P65, p-P38, p-ERK, p-JNK, and p-AKT, thereby alleviating UC [143]. At the same time, SONG J et al. reported that honey polysaccharides (AHPN50-1a) with repeated (1→6) -α-GlcP structures can downregulate the expression levels of IL-1β, IL-6, and TNF-α in mouse colon tissue and restore intestinal microbial diversity and SCFA concentrations, thus reducing intestinal inflammation in mice [173]. CHENG Y et al. reported that *Poria cocos* polysaccharide (PCP-1C), which has a 1,3-β-D-Glcp structure, could significantly reduce the release of IL-1β, IL-6, and TNF-α and increase the contents of SOD and GSH-Px, thereby ameliorating CCl4-induced liver injury in mice [146]. In addition, *Ganoderma lucidum* polysaccharide (MBG), which has a β-1→3 and β-1→6 glucan structure, can reduce the number of inflammatory cells in the heart, liver, kidney, spleen, and other tissues of mice fed a high-cholesterol diet and induce the production of serum IgA and IgG. Increased expression of the poly-Ig receptor in the small intestine and increased IL-2 production in NK cells were observed [174]. Liu Wenjun reported that *Angelica sinensis* polysaccharide (APS-2I) with an α-D-β-Galp-(1→6) structure can significantly reduce the level of MyD88 in the medium of LPS-induced macrophages and in the serum of mice with sepsis and inhibit the formation of the TLR4 and MD-2 complex and the increases in TNF-α, IFN-β, IL-6, and NO levels [139]. Carboxymethyl *Poria cocos* polysaccharide (CMP44) is a homogeneous polysaccharide with a main chain structure of (1→3) -β-d-glucan, a small amount of (1→6)-β and (1→2)-β glucoside bonds, and a triple helix structure. The experimental results showed that CMP44 inhibits the release of NO, TNF-α, IL-6, and IL-1β from RAW264.7 cells exposed to LPS to varying degrees, thus exerting anti-inflammatory effects [147]. In addition, *Ganoderma lucidum* polysaccharide (BSGLP), which has a (1→3)-β-D-Glcp and (1→6)-β-D-Glcp structure, can effectively relieve the inflammation induced by an HFD in mice by inhibiting the upregulation of the TLR4/Myd88/NF-κB signaling pathway [36]. A summary is given in Table 5.

These results suggest that α/β-(1→3), (1→6) glucoside linkages may be the key to the anti-inflammatory activity of MEHTCMPs, among which β-(1→3), (1→6) glucoside linkages are more prominent.

### 3.2. Advanced Structure

The advanced structure of polysaccharides is based on the primary structure, and the complex advanced structure is formed by hydrogen bonding or noncovalent bonding interactions between the backbone chains. Due to the different compositions of monosaccharides and glycoside linkages, polysaccharide molecules in solution have various conformations, such as irregular cluster chains, single helices, double helices, triple helices, and wormlike structures [175]. Among them, the triple helix polysaccharide is particularly attractive, and the triple helix structure can confer greater biological activity on the polysaccharide [176]. Studies have shown that the triple helix structure of carboxymethyl *Poria cocos* polysaccharide (CMP33) can significantly inhibit the release of IL-6, TNF-α, and IL-1β from RAW264.7 cells stimulated with LPS, with the maximum inhibition rates reaching 48.0%, 79.7%, and 51.8%, respectively [148]. With decreasing molecular weight, *Ganoderma lucidum* polysaccharide (GLP), which also has a triple helix structure, can significantly reduce the expression of TNF-α, IL-1β, and IL-6 in the colon tissues of mice with DSS-induced ulcerative colitis and enhance the inhibition of L-selectin and ligand binding [135].

In addition, the analysis of physical characteristics is also one of the key steps in polysaccharide analysis. The analysis of the appearance characteristics of polysaccharides can help to determine the relationship between the appearance characteristics and biological activity of a polysaccharide to identify polysaccharides with biological activity. Using scanning electron microscopy, LI Q et al. reported that *Pueraria montana var. thomsonii* polysaccharide (RPP-2), which has a smooth, clean, and irregular sheet structure, can significantly reduce the level of TNF-α in the serum of HFD-induced NAFLD mice and alleviate liver inflammation [170]. FANG S et al. reported that *Gardenia jasminoides* polysaccharide (GPS), which has a large number of irregular, thin, randomly distributed, and amorphous structures, can significantly reduce the expression of TLR4, NF-κB, and MyD88, as well as the levels of MCP-1 and IL-6, in the livers of cholestatic mice, thus alleviating cholestatic liver injury [177]. In addition, studies have shown that the carboxymethyl *Pseudocydonia sinensis* polysaccharide CSP-M, which has a sheet surface and is accompanied by many porous structures, can significantly reduce the infiltration of inflammatory cells in the colon tissue of mice; reduce the levels of MPO, TNF-α, IL-1β, IL-6, NO, and MDA; and improve the activities of SOD and GSH, which can effectively relieve the symptoms of UC [178]. Other studies have shown that the GP-Zn(II) complex between *ginger* polysaccharide and iron, which has a flat surface, a sheet structure, and partial dendritic fragments can significantly reduce the expression levels of the IL-1β, IL-6, IL-8, IL-12, and TNF-α mRNAs in zebrafish and upregulate the expression of IL-10, reducing CUSO4-induced inflammation [179]. A summary is given in Table 6.

According to the aforementioned analysis of appearance characteristics, although different MEHTCMPs have slightly different appearance characteristics, most of them have a flaky structure, which indicates that a flaky structure may be one of the factors influencing the anti-inflammatory activity of MEHTCMPs.

### 3.3. Structural Modification

Polysaccharides are macromolecules with biological activity, but some polysaccharides have no biological activity or have low biological activity because of their structures. Studies have shown that structural modifications, such as changes in molecular weight [180], monosaccharide composition [181], spatial structure [182,183], and physical characteristics [184,185], can change the structure of a compound. Therefore, structural modification of polysaccharides is often performed to change these factors and improve their biological activity [186,187]; thus, polysaccharides have wider applications in biomedicine. Among them, chemical modification is the most commonly used method [188]. These reactions can be divided into sulfation, phosphorylation, acetylation, carboxymethylation, selenization, and other processes.

#### 3.3.1. Selenization

**Figure 7:**
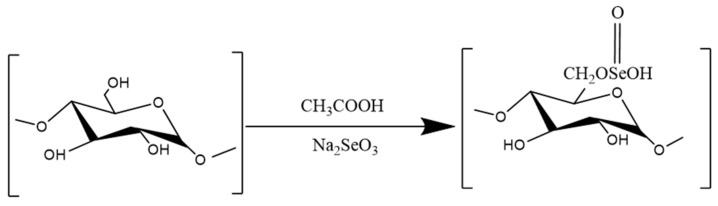
Selenization modification of polysaccharides.

Selenization is an effective method for introducing selenium into polysaccharides and enhancing their biological activities. As shown in Figure 7. Polysaccharides containing selenide can play multiple roles in polysaccharide and selenium functions, and their activity is much higher than that of selenium or polysaccharides, which is more conducive to their absorption and utilization by the body [189], with lower toxicity and higher bioavailability [190]. A summary is given in Table 7.

Zhu Xiaoqing et al. reported that compared with GPS, selenated *Glycyrrhiza uralensis* polysaccharide (Se-GPS) significantly reduced the release of TNF-α and IL-1β in mouse serum [191]. The study by HAMID M showed that selenated *Astragalus membranaceus* polysaccharide (sAPS3) could significantly reduce the production of IL-1β and TNF-α, thereby alleviating CCL4-induced hepatocyte necrosis and inflammation [130]. In addition, YE R et al. reported that EUP-SeNP, a complex of 170 nM *Eucommia ulmoides* polysaccharides and selenium nanoparticles, could significantly reduce the release of IL-1β, IL-6, IL-12, IL-17, and TNF-α in mice with DSS-induced colitis and in LPS-stimulated IEC-6 and Caco-2 cells. The expression of P-IκB/IκB, p-p65/p65, and TLR-4 was also decreased, and the content of IL-10 was increased; thus, this polysaccharide exerted an anticolitic effect [192]. In addition, GAO Z et al. reported that the selenated *Angelica sinensis* polysaccharide sCAP can significantly increase the total phosphorus (TP) content in the serum of mice; decrease the contents of ALT, AST, ALP, and MDA and ROS in liver tissue; and increase the activities of SOD and T-AOC. The expression of the p-ERK, p-JNK, and p-p38 proteins was significantly inhibited, thereby alleviating CCI4-induced liver injury [140].

#### 3.3.2. Carboxymethylation

**Figure 8:**
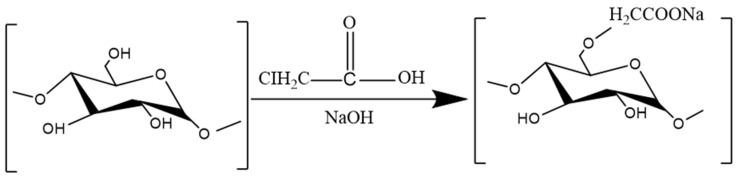
Carboxymethylation of polysaccharides.

Carboxymethylation refers to the substitution of certain hydroxyl groups on polysaccharide residues with carboxymethyl groups [193]. As shown in Figure 8. After the carboxymethyl modification, the conformation of the polysaccharide can be changed, and the biological activity can be improved [194,195]. A summary is given in Table 7.

Studies have shown that *Poria cocos* polysaccharides can be divided into water-soluble and alkali-soluble components according to their solubility. However, the content of water-soluble polysaccharide, which is the main pharmacologically active component of *Poria cocos*, is extremely low, usually ranging from 0.7% to 2.6% [196]. In contrast, the percentage of alkali-soluble polysaccharides with low pharmacological activity is as high as 70% to 90% [197,198]. This composition severely limits the development and utilization of *Poria cocos*. Therefore, carboxymethylation is usually used to modify *Poria cocos* to improve its pharmacological activity.

LIU X et al. reported that carboxymethyl *Poria cocos* polysaccharide (CMP33), which has a molecular weight of 52.3 kDa, could significantly reduce the LPS-induced secretion of NO, IL-1β, IL-6, and TNF-α from RAW264.7 cells and had good anti-inflammatory effects [148]. Similarly, LI C et al. reported that carboxymethylation can change the surface microstructure of *Pseudocydonia sinensis* polysaccharide (CSP), making it appear flaked and accompanied by many porous structures. This structure is conducive to the absorption and utilization of polysaccharides, thus improving the anti-inflammatory activity of CSP. It can significantly reduce the release of the inflammatory factors TNF-α, IL-1β, and IL-6 in the colon tissue of UC mice. This result suggests that carboxymethylation may be an effective method for enhancing the biological activity of CSP [178]. Li Yawei et al. reported that carboxymethyl *Ganoderma lucidum* polysaccharide (CM-GLP) can effectively reduce the expression of NF-κB, TNF-α, IL-1, and IL-6 in rat brain tissue; reduce the inflammatory response; and thus alleviate cerebral ischemia-reperfusion injury in rats [134].

#### 3.3.3. Sulfation

**Figure 9:**
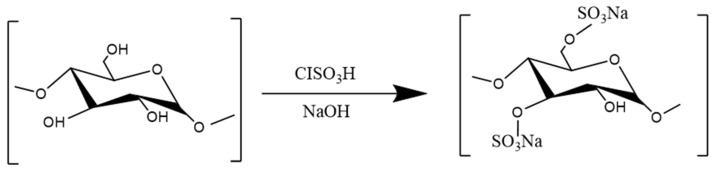
Sulfation modification of polysaccharides.

Sulfation refers to the introduction of sulfuric acid groups into some hydroxyl groups of polysaccharide chains. As shown in Figure 9. This method can effectively change the water solubility and biological activity of polysaccharides, and thus it is widely used to modify polysaccharide molecules [199,200]. A summary is given in Table 7.

Studies have shown that sulfation can enhance the anti-inflammatory activity of polysaccharides. WANG X et al. showed that sulfated *Astragalus membranaceus* polysaccharide (SAPS) could significantly downregulate the expression of the TNF-α, IL-1β, IL-8, and TLR4 mRNAs in LPS-induced Caco2 cells and reduce the expression of ZO-1, showing better anti-inflammatory activity than the polysaccharide without the modification [131]. Qiang-Ming Li et al. isolated three sulfated *Laminaria japonica* polysaccharides (SLJP1, SLJP2, and SLJP3) from *Laminaria japonica* polysaccharides (LJP61A). The results showed that all three *Laminaria japonica* polysaccharides blocked OX-LDL-induced PPAR-γ activation in macrophages. The release of TNF-α, IL-1β, and IL-6 was reduced, and the degree of inhibition was proportional to the degree of polysaccharide sulfation [201,202]. Sulfation may be an important means to enhance the anti-inflammatory activity of *Laminaria japonica* polysaccharides. SONG X et al. reported that *Ganoderma lucidum* polysaccharide (SGRP), which has a 7.8% sulfur content, could inhibit the activation of the TLR4/NF-κB signaling pathway and significantly reduce the levels of the inflammatory factors TNF-α, IL-1β, and IL-6 in mouse liver tissue, thus alleviating CCl4-induced chronic liver injury in mice [132]. Liu Yanfang obtained a series of *Ganoderma lucidum* polysaccharide (GLP) derivatives with different substitution degrees and molecular weights by sulfation. The results showed that sulfation could change the molecular weight and conformational characteristics of GLP and significantly inhibit the release of NO induced by LPS. Moreover, the greater the degree of sulfate group substitution, the greater the anti-inflammatory activity [135].

#### 3.3.4. Complexation with Metal Ions

In recent years, an increasing number of reports have described the complexes formed by polysaccharides and metal ions and their biological activities, and studies have shown that the synergistic interaction between polysaccharides and metal ions can enhance the biological activities of polysaccharides [203,204,205]. A summary is given in Table 7.

**Table 7:** Structural Modification of MEHTCMPs with anti-inflammatory effect.

Source	Compound Name	Structural Modification	Effects	References
*Glycyrrhiza uralensis*	Se-GPS	Selenization	TNF-α ↓, IL-1β ↓	[191]
*Astragalus membranaceus*	sAPS3	Selenization	TNF-α ↓, IL-1β ↓	[130]
*Eucommia ulmoides*	EUP-SeNP	Selenization	IL-1β ↓, IL-6 ↓, IL-12 ↓, IL-17 ↓, TNF-α ↓, P-IκB/IκB ↓, p-p65/p65 ↓, TLR-4 ↓, IL-10 ↑	[192]
*Angelica sinensis*	sCAP	Selenization	TP ↑, SOD ↑, T-AOC ↓, ALT ↓, AST ↓, ALP ↓, MDA ↓, ROS ↓, p-ERK ↓, p-JNK ↓, p-p38 ↓	[140]
*Poria cocos*	CMP33	Carboxymethylation	NO ↓, IL-1β ↓, IL-6 ↓, TNF-α ↓	[148]
*Pseudocydonia sinensis*	CSP	Carboxymethylation	TNF-α ↓, IL-1β ↓, IL-6 ↓	[178]
*Ganoderma lucidum*	CM-GLP	Carboxymethylation	NF-κB ↓, TNF-α ↓, IL-1 ↓, IL-6 ↓	[134]
*Astragalus membranaceus*	SAPS	Sulfation	TNF-α ↓, IL-1β ↓, IL-8 ↓, TLR4	[131]
*Laminaria japonica*	SLJP1	Sulfation	TNF-α ↓, IL-1β ↓, IL-6 ↓, PPAR-γ ↓	[201,202]
*Laminaria japonica*	SLJP2	Sulfation	TNF-α ↓, IL-1β ↓, IL-6 ↓, PPAR-γ ↓	[201,202]
*Laminaria japonica*	SLJP3	Sulfation	TNF-α ↓, IL-1β ↓, IL-6 ↓, PPAR-γ ↓	[201,202]
*Ganoderma lucidum*	SGRP	Sulfation	TNF-α ↓, IL-1β ↓, IL-6 ↓, TLR4 ↓, NF-κB ↓	[132]
*Ganoderma lucidum*	GLP	Sulfation	NO ↓	[135]
*ginger*	GP-Zn(II)	Introduce Zn	IL-1β ↓, IL-6 ↓, IL-8 ↓, IL-12 ↓, TNF-α ↓, IL-10 ↑	[179]
*Laminarin*	LP-SR	Introduce SR	IL-6 ↓	[206]
*Eucommia ulmoides*	EUP-Sr	Introduce SR	IL-1β ↓	[207]

LI W et al. complexed zinc with *ginger* polysaccharide (GP) to produce GP-Zn(II) containing 21.17 mg/g zinc and reported that the introduction of Zn not only reduced the crystallinity and asymmetry of GPs, but also changed its appearance to a compact, relatively flat surface with a lamellar structure and some dendritic fragments. The introduction of Zn into GPs can change its structure, which may be beneficial to its anti-inflammatory activity; significantly downregulate IL-1β, IL-6, IL-8, IL-12, and TNF-α mRNA expression; and upregulate the level of IL-10 in CusO4-induced zebrafish [179]. In addition, MA F et al. successfully synthesized a strontium-complexed *Laminarin* polysaccharide (LP-SR), and the results showed that LP-SR not only had a better thermal stability than LP but also significantly reduced the release of IL-6 from HUVECs and MC3T3-E1 cells, indicating that the introduction of strontium can effectively improve the anti-inflammatory activity of LP. It can be applied to the development of bone repair biomaterials or devices [206]. At the same time, DENG et al. reported that the introduction of Sr not only improved the thermal stability of *Eucommia ulmoides* polysaccharide (EUP) but also optimized the disordered structure of EUP, decreasing its particle size and increasing its uniformity. This change may be related to EUP-Sr inhibiting the activation of the NF-κB signaling pathway in RAW264.7 cells and reducing the level of IL-1β [207]. Therefore, based on this information, MENGDI et al. introduced EUP-Sr onto a PEEK surface to generate a novel DPEEK@EUP-Sr complex. The biological activity of the complex was investigated. The results showed that DPEEK@EUP-Sr effectively promoted the proliferation of preosteogenic MC3T3-E1 cells. The expression of RUNX2 and Col1-α1 was significantly upregulated, and the expression of IL-1β, IL-18, and MMP9 was downregulated; these factors have significant anti-inflammatory and osteogenic effects and are potential bone repair agents with dual effects on inflammation and bone formation [208].

The above studies showed that chemical modification can significantly improve the anti-inflammatory activities of MEHTCMPs, as manifested by changes in the physical and chemical properties of MEHTCMPs, such as their molecular weight and solubility. This approach is an effective method to develop and utilize MEHTCMPs in the future. However, deficiencies in the research on chemically modified MEHTCMPs still exist. (1) Few studies have evaluated the toxicity of MEHTCMPs after modification, and systematic studies on the toxicological properties of MEHTCMPs before and after modification are urgently needed. (2) The structure and activity mechanism of chemically modified MEHTCMPs still needs to be studied further. (3) Different chemical modification methods and conditions will produce different products. The current modification methods for MEHTCMPs have certain limitations, and researchers need to constantly improve the modification methods to obtain the ideal modified products. (4) Because chemical modification requires the consumption of a certain amount of organic reagents, chemical modification can pollute the environment. Therefore, in future research on the anti-inflammatory activities of MEHTCMPs, researchers should not only pay attention to structural modifications but also to the toxicity of modified MEHTCMPs and further optimize the modification methods to reduce environmental pollution.

Although many researchers have performed a series of studies and analyses on the structure of MEHTCMPs, most of them still focus on the primary structure, and analyses on the advanced structure of MEHTCMPs are limited. The relationship between the primary structures and advanced structures of MEHTCMPs and their anti-inflammatory activities is still unclear. The relationship between the primary structures and higher-order structures and the pharmacological activities of MEHTCMPs is still the focus and trend of future research. In the future, studies on the relationship between the anti-inflammatory activity and structure–efficacy relationship of MEHTCMPs can focus on the following aspects: for example, determining the specific molecular weight range in which different MEHTCMPs exert their anti-inflammatory activity and exploring the anti-inflammatory mode of MEHTCMPs fragments in this range, whether they directly act on the immune system or indirectly act on intestinal microorganisms. The configuration and conformation of the monosaccharides and sugar chains of the anti-inflammatory MEHTCMPs were studied to reveal the structures necessary for their anti-inflammatory activities. Based on this information, the necessary structural modification of MEHTCMPs was performed to increase or decrease the number of members of a certain group to maximize the anti-inflammatory activity of MEHTCMPs. By studying and solving the above problems, research on the anti-inflammatory activity of MEHTCMPs will definitely be a qualitative improvement.

## 4. Conclusions and Future Prospects

Along with our country’s economic development and social progress, the pursuit of health has become one of the topics of greatest concern. The Outline of the Healthy China 2030 Plan has set the goals of “significantly improving the physical fitness of the people, reaching 79.0 years of average life expectancy by 2030, and significantly increasing average healthy life expectancy” and “greatly improving the health literacy of the whole people, and fully promoting a healthy lifestyle.” People have taken traditional Chinese medicine as the main means of health care, and MEHTCMPs have both the efficacy attributes of traditional Chinese medicine and food attributes and are effective approaches to improve the health quality of the whole population, which can serve the concept of a “healthy China.” Therefore, the country is paying increasing attention to the development of the medicinal and edible homologues industry, and the development of medicinal and edible homologous traditional Chinese medicine has become an emerging research direction in the fields of medicine and food. As one of the important active components of MEHTCMPs, polysaccharides have become a hot topic in the fields of biochemistry and molecular biology, after proteins and nucleic acids. Drawing on modern developed science and technology, a large number of researchers have performed the effective extraction, separation and purification, and structural analysis of MEHTCMPs to promote research on their functional components, such as structure–activity relationships, dose–effect relationships, and action mechanisms, and to develop products using MEHTCMPs as raw materials with clear mechanisms, clear effects, safety, and stability. The unique advantages of ethnic medicine in preventing and treating diseases should be fully considered.

In recent years, an increasing number of MEHTCMPs have been reported to have anti-inflammatory effects, and the key anti-inflammatory signaling pathways involved have been identified. Their molecular mechanisms of action are mainly related to the regulation of signaling pathways such as the TLR, MAPK, JAK/STAT, PI3K/AKT, Nrf2/HO-1, PPAR-γ, and NLRP3 pathways. These signaling pathways have become the main sources of new anti-inflammatory drug targets. Moreover, the relationship between structure and efficacy can be clarified by analyzing the structures of MEHTCMPs, and the structures of MEHTCMPs can be changed by structural modification to increase or decrease the anti-inflammatory activity. These findings provide further evidence for the anti-inflammatory effects of MEHTCMPs and indicate that MEHTCMPs have the potential to be used as lead compounds for the development of new anti-inflammatory drugs. However, although some progress has been made in understanding the anti-inflammatory activity of MEHTCMPs, many challenges in the study of MEHTCMPs remain. First, polysaccharides are the most complex polymers in nature, and their structures can be divided into primary, secondary, tertiary, and quaternary structures. Although chemical structures of polysaccharides have been characterized through chemical and instrumental analyses, several difficulties exist, such as their complexity, time consumption, and inability to perform microanalysis. As a result, the chemical structures of many MEHTCMPs have not been clearly characterized. Even if the chemical structures of some MEHTCMPs have been characterized, most of them are limited to the primary structures. The difficulty in structural characterization based on the unusual complexity of the structures of MEHTCMPs has led to structural modification and synthesis, and subsequent studies of structure–activity relationships are still challenging. In addition, oral administration is the most common method of drug delivery for MEHTCMPs, which are characterized by a large relative molecular mass, strong hydrophilicity, multiple electrical charges, and poor stability, and thus MEHTCMPs are not easily absorbed into the bloodstream through intestinal epithelial cells. Therefore, a clear understanding of how MEHTCMPs are absorbed and utilized after oral administration, whether they are absorbed in the prototypic form or in the degraded form, and to what extent they are absorbed, distributed, metabolized, excreted, and transformed is still lacking. In addition, some studies using in vitro models to evaluate the activity of MEHTCMPs encounter the problem of directly adding MEHTCMPs to cell models to evaluate and screen the activity of MEHTCMPs. Since the absorption and metabolism of MEHTCMPs are unknown, whether MEHTCMPs enter the body in the form of prototypes or metabolites and whether they can contact target cells are unclear. False-positive or false-negative results can easily be produced. Therefore, the establishment of activity evaluation models consistent with the biological characteristics of MEHTCMPs, including animal models, tissue models, cell models, receptor models, computer virtual models, etc., must be clear on the premise of MEHTCMPs absorption and metabolism pathways to more reliably evaluate MEHTCMPs’ activity and fully elucidate the pharmacological mechanism of MEHTCMPs. Clarifying MEHTCMPs’ absorption and metabolic pathways is also an issue on which future researchers must focus. Although researchers have made many efforts to determine the relationship between the structure and anti-inflammatory activity of MEHTCMPs, due to technical limitations, this relationship remains unclear, and researchers need to continue to pay attention to and improve structural analysis and modification methods to further reveal the deeper links between them. In the future, with the continuous development of modern chemistry and biology and other technologies, clinical studies on the anti-inflammatory mechanism of MEHTCMPs should be combined with modern scientific methods and regulatory methods to further conduct in-depth studies on the material basis, pharmacological mechanism of action, toxicological evaluation, and relationship between structure and efficacy. Research on the anti-inflammatory activity and product development of MEHTCMPs will certainly reveal broader application prospects.

## Figures and Tables

**Table 1:** A total of 110 species of MEHTCMs.

Number	Chinese Name	English Name	Latin Name	Name of Family	Part Used
1	Baibiandou	Semen Dolichoris Album	*Dolichos lablab* L.	Fabaceae	Mature seed
2	Baibiandouhua	Flower of Hyacinth Dolichos	*Dolichos lablab* L.	Fabaceae	Flower
3	Baiguo	ginkgo seed	*Ginkgo biloba* L	Ginkgoaceae	Mature seed
4	Baihe	lily	*Lilium lancifolium* Thunb. *Lilium brownie* F.E.Brown var.viridulum Baker *Lilium pumilum* DC.	Liliaceae	Fleshly scale leaf
5	Baimaogen	rhizoma imperatae	*Imperata cylindrica* Beauv.var. major (Nees) C.E.Hubb.	Poaceae Barnhart	Rhizome
6	Baizhi	angelica	*Angelica dahurica* (Fisch.ex Hoffm.) Benth.et Hook.f *Angelica dahurica* (Fisch.ex Hoffm.) Benth. et Hook.f.var.*formosana* (Boiss.) Shan et Yuan	Apiaceae	Root
7	Bajiaohuixiang	Anisi Stellati Fructus	*Illicium verum* Hook.f.	Magnoliaceae	Ripe fruit
8	Biba	long pepper	*Piper longum* L.	Piperaceae Giseke	Fruit/ripe ear
9	Bohe	mint	*Mentha canadensis* L.	Lamiaceae	Overground part
10	Buzhaye	leaf of paniculate microcos	*Microcos paniculata* L.	Tiliaceae	Leaf
11	Caoguo	Amomum tsao-ko	*Amomum tsao-ko* Crevost et Lemaire	zingiberaceae	Fruit
12	Chenpi	dried tangerine peel	*Citrus reticulata* Blanco	Rutaceae	Ripe peel
13	Chixiaodou	ricebean	*Vigna umbellate* (Thunb.) Ohwi & Ohashi	Fabaceae	Mature seed
14	Daidaihua	seville orange flower	*Citrus aurantium* L.var.amara Engl.	Rutaceae	Flower bud
15	Dandouchi	fermented soybean	*Glycine max* (L.) Merr.	Fabaceae	Mature seeds
16	Danggui	*Angelica sinensis*	*Angelica sinensis* (Oliv.) Diels	Apiaceae	Root
17	Dangshen	*Salvia miltiorrhiza*	*Codonopsis pilosula* (Franch.) Nannf.	Campanulaceae	Root
18	Danzhuye	*Lophatherum gracile*	*Lophatherum gracile* Brongn.	Poaceae Barnhart	Stem leaf
19	Daodou	blade bean	*Canavalia gladiate* (Jacq.) DC	Fabaceae	Mature seed
20	Dingxiang	clove	*Eugenia caryophyllata* Thunb	Myrtaceae	Bud
21	Duzhongye	folium cortex eucommiae	*Eucommia ulmoides* Oliv.	Eucommiaceae	Leaf
22	Ejiao	donkey-hide gelatin	*Equus asinus* L.	Equidae	skin
23	Feizi	Chinese torreya	*Torreya grandis* Fort.	Taxaceae Gray	Mature seed
24	Fenge	Pueraria kudzu	*Pueraria montana var. thomsonii* (Benth.) Wiersema ex D. B. Ward	Fabaceae	Root
25	Fengmi	honey	*Apis cerana* Fabricius	Apoidea	Nectar, secreta
26	Foshou	fingered citron	*Citrus medica* L.var.sarcodactylis Swingle	Rutaceae	Fruit
27	Fuling	Poria cocos	*Poria cocos*(Schw.)Wolf	Polyporaceae	Sclerotium
28	Fupenzi	raspberry	*Rubus chingii* Hu	Rosaceae	Fruit
29	Gaoliangjiang	Alpinia officinarum	*Alpinia officinarum* Hance	zingiberaceae	Rhizom
30	Gegen	lobed Kudzuvine root	*Puerariae Lobatae* Radix	Fabaceae	Root
31	Gouqizi	Chinese wolfberry	*Lycium chinense* Miller	Solanaceae	Ripe fruit
32	Gancao	liquorice root	*Glycyrrhiza uralensis* Fisch. *Glycyrrhiza inflata* Bat. *Glycyrrhiza glabra* L	Fabaceae	Root/rhizome
33	Heihujiao	black pepper	*Piper nigrum* L.	Piperaceae Giseke	Near ripe/ripe fruit
34	Heizhima	Semen sesami nigrum	*Sesamum indicum* L	Pedaliaceae	Mature seed
35	Heye	lotus leaf	*Nelumbo nucifera* Gaertn.	Nymphaeaceae	Leaf
36	Huaihua	Sophora flower	*Sophora japonica* Linn	Fabaceae	Flower
37	Huaimi	Sophora flower-bud	*Sophora japonica* Linn	Fabaceae	Flower bud
38	Huajiao	Sichuan pepper	*Zanthoxylum bungeanum* Maxim.	Rutaceae	Ripe peel
39	Huangjiezi	yellow mustard	*Brassica juncea* (L.) Czern.et Coss	Brassicaceae	Mature seed
40	Huangjing	rhizoma polygonati	*Polygonatum kingianum* Coll.et Hemsl. *Polygonatum sibiricum* Red. *Polygonatum cyrtonema* Hua	Liliaceae	Rhizome
41	Huangqi	milk vetch root	*Astragalus membranaceus* (Fisch.) Bunge	Fabaceae	Root
42	Huomaren	Semen Cannabis	*Cannabis sativa* L.	Moraceae	Ripe fruit
43	Huoxiang	Agastache rugosus	*Agastache rugosa* (Fisch. & C. A. Mey.) Kuntze	Lamiaceae	Overground part
44	Jiang	ginger	*Zingiber officinale* Roscoe	zingiberaceae	Rhizom
45	Jianghuang	turmeric	*Curcuma longa* L.	zingiberaceae	Rhizome
46	Jiegeng	Platycodon grandiflorus	*Platycodon grandifloras* (Jacq.) A.DC.	Campanulaceae	Root
47	Jineijin	endothelium corneum gigeriae galli	*Gallusgallusdomesticus* Brisson	Phasianidae	Inner wall of gizzard
48	Jinyinhua	honeysuckle	*Lonicera japonica* Thunb.	Caprifoliaceae	Buds/budding Flowers
49	Juemingnzi	Cassia seed	*Cassia obtusifolia* L. *Cassia tora* L.	Fabaceae	Mature seed
50	Juhong	exocarpium	*Citrus reticulata* Blanco	Rutaceae	Outer peel
51	Juhua	chrysanthemum	*Chrysanthemum morifolium* Ramat	Asteraceae	Capitulum
52	Juju	witloof	*Cichorium intybus* L.	Asteraceae	Anaerial part/root
53	Kunbu	kombucha	*Ecklonia kurome* Okam. *Laminaria japonica* Aresch.	Laminariaceae	Thallus
54	Laifuzi	radish seed	*Raphanus sativus* L.	Brassicaceae	Mature seed
55	Lianzi	lotus seed	*Nelumbo nucifera* Gaertn.	Nymphaeaceae	Mature seed
56	Lingzhi	*Ganoderma lucidum*	*Ganoderma lucidum* (Curtis) P. Karst.	Polyporaceae	Fruiting body
57	Longyanrou	longan flesh	*Dimocarpus lon.gan* Lour.	Sapindaceae	Aril
58	Lugen	rhizoma phragmitis	*Phragmites communis* Trin.	Poaceae Barnhart	Rhizome
59	Luohanguo	Momordica grosvenori	*Siraitia grosvenorii* (Swingle) C. Jeffrey ex Lu et Z. Y. Zhang	Cucurbitaceae	Fruit
60	Machixian	purslane	*Portulaca oleracea* L	Portulacaceae	Overground part
61	Maiya	malt	*Hordeum vulgare* L.	Poaceae Barnhart	Ripe fruit
62	Meiguihua	rose	*Rosa rugosa* Thunb *or Rose rugosa* cv. Plena	Rosaceae	Flower bud
63	Mugua	pawpaw	*Chaenomeles speciosa* (Sweet) Nakai	Rosaceae	Near ripe fruit
64	Muli	oyster	*Ostreidae*	Ostreidae	Shell
65	Pangdahai	sterculia scaphigera	*Sterculia lychnophora* Hance	Sterculiaceae	Mature seed
66	Pugongying	dandelion	*Taraxacum mongolicum* Hand.-Mazz.	Asteraceae	Whole herb
67	Qianshi	Semen Euryales	*Euryale ferox* Salisb. ex Konig et Sims	Nymphaeaceae	Mature seed kernel
68	Qingguo	Chinese white olive	*Canarium album* Raeusch	Burseraceae	Ripe fruit
69	Qishe	long-noded pit viper	*Agkistrodon acutus* (Guenther)	Viperidae	Dried body
70	Renshen	ginseng	*Panax ginseng* C. A. Mey.	Araliaceae	Root/rhizome
71	Roucongrong	cistanche	*Cistanche deserticola* Ma	Orobanchaceae	Succulent stem
72	Roudoukou	myristica fragrans	*Myristica fragrans* Houtt.	Myristicaceae	Kernel/seed coat
73	Rougui	cinnamon	*Cinnamomum cassia* Presl	Lauraceae	Bark
74	Sangshen	mulberry	*Morus alba* L.	Moraceae	Ruit ear
75	Sangye	folium mori	*Morus alba* L.	Moraceae	Leaf
76	Shaji	sea-buckthorn	*Hippophae rhamnoidese* L.	Elaeagnaceae	Ripe fruit
77	Shannai	rhizoma kaempferiae	*Kaempferia galanga* L.	zingiberaceae	Rhizome
78	Shanyao	Chinese yam	*Dioscorea opposita* Thunb.	Dioscoreaceae	Rhizome
79	Shanyinhua	lonicerae flos	*Lonicera macranthoides* Hand.-Mazz	Caprifoliaceae	Buds/budding Flowers
80	Shanzha	hawthorn	*Crataegus pinnatifida* Bge.var.major N.E.Br. *Crataegus pinnatifida* Bge.	Rosaceae	Ripe fruit
81	Shanzhuyu	dogwood	*Cornus officinalis Sieb. et* Zucc.	Cornaceae	Fruit
82	Sharen	fructus amomi	*Amomum villosum* Lour.var.*xanthioides* T.L.Wu et Senjen	zingiberaceae	Ripe fruit
83	Songhuafen	pollen pini	*Pinus massoniana* Lamb.	Pinaceae	Dried pollen
84	Suanzaoren	spina date seed	*Ziziphus jujuba* Mill.var.spinosa (Bunge) Hu ex H.F.Chou	Rhamnaceae	Pulp/mature seeds
85	Taoren	peach kernel	*Prunus persica* (L.) Batsch *Prunus davidiana* (Carr.) Franch.	Rosaceae	Mature seed
86	Tianma	gastrodia elata	*Gastrodia elata* Bl.	Orchidaceae	Tuber
87	Tiepishihu	Dendrobium officinale	*Dendrobium officinale Kimura &* Migo	Orchidaceae	Stem
88	Wumei	black plum	*Prunus mume* (Sieb.) Sieb.et Zucc	Rosaceae	Near ripe fruit
89	Wushaoshe	zaocys dhumnade	*Zaocys dhumnades*	Colubridae	Dried body
90	Xiakucao	selfheal	*Prunella vulgaris* L.	Lamiaceae	Fruit ear
91	Xiangru	elsholtzia	*Elsholtzia ciliata* (Thunb.) Hyl.	Lamiaceae	Overground part
92	Xiangyuan	citron	*Citrus medica* L.	Rutaceae	Ripe fruit
93	Xiaohuixiang	fennel	*Foeniculum vulgare* Mill.	Apiaceae	Ripe fruit
94	Xiaoji	artichoke	*Cirsium setosum* (Willd.) MB.	Asteraceae	Overground part
95	Xiebai	allium macrostemon	*Allium macrostemon* Bunge	Liliaceae	Bulb
96	Xihonghua	stigma croci	*Crocus sativus* L	Iridaceae	Stigma
97	Xingren	almond	*Prunus armeniaca* L.var.ansu Maxim *Prunus sibirica* L. *Prunus mandshurica* (Maxim) Koehne *Prunus armeniaca* L.	Rosaceae	Mature seed
98	Xiyangshen	American ginseng	*Panax quinquefoliu* L.	Araliaceae	Root/rhizome
99	Yansui	coriander	*Coriandrum sativum* L.	Apiaceae	Fruit/seed
100	Yiyiren	semen coicis	*Coix lacryma-jobi* L.var.mayuen (Roman.) Stapf	Poaceae Barnhart	Mature seed kernel
101	Yizhiren	fructus Alpiniae oxyphyllae	*Alpinia oxyphylla* Miq.	zingiberaceae	Nuts/fruit
102	Yuganzi	emblic leafflower fruit	*Phyllanthus emblica* L.	Euphorbiaceae	Ripe fruit
103	Yuliren	bunge cherry seed	*Prunus humilis* Bge. *Prunus japonica* Thunb. *Prunus pedunculata* Maxim.	Rosaceae	Mature seed
104	Yuxingcao	fish mint	*Houttuynia cordata* Thunb.	Saururaceae	Whole grass/ground parts
105	Yuzhu	radix polygonati officinalis	*Polygonatum odoratum* (Mill.) Druce	Liliaceae	Rhizome
106	Zao	jujube	*Ziziphus jujuba* Mill.	Rhamnaceae	Ripe fruit
107	Zhijuzi	Turnjujube	*Hovenia dulcis* Thunb.	Rhamnaceae	Rachis, leaves, and stem branches
108	Zhizi	Cape jasmine	*Gardenia jasminoides* J.Ellis	Rubiaceae	Ripe fruit
109	Zisu	purple perilla	*Perilla frutescens* (L.) Britt.	Lamiaceae	Leaf/twigs
110	Zisuzi	perilla seed	*Perilla frutescens* (L.) Brit	Lamiaceae	Ripe fruit

**Table 2:** Anti-inflammatory inhibitory effects of different MEHTCMPs in different cell/animal models.

Source	Compound Name	Model	Dose	Molecular Weight	Monosaccharide Composition and Ratio	Glycosidic Bond	Effects	Mechanisms	References
*Astragalus membranaceus*	APS	IPEC-J2 cell BALB/c mice (LPS-induced inflammation model)	0.2 mL 200 mg/kg 7 days				p-p38 ↓, ERK1/2 ↓, IκB-α ↑, IL-6 ↓, IL-1α ↓, TNF-α ↓, IL-1β ↓, CXCL8 ↓, TNFAIP3 ↓, CXCL2 ↓, BCL3 ↓, BNIP3 ↓	Alleviating LPS-induced inflammation by inhibiting the MAPK and NF-κB signaling pathways	[57]
*Astragalus membranaceu*	APS-I	RAW264.7 cell (LPS-induced inflammation model)	10, 25, 50, 100 μg/mL	>2000 kDa	Man, Rha, GalA, Glu, Gal, Ara 0.54∶0.26∶12.24∶17.24∶8.46∶1		NO ↓, TNF-α ↓, IL-10 ↑	Closely related to amino acid metabolism and energy metabolism	[127]
*Astragalus membranaceu*	APS-II	RAW264.7 cell (LPS-induced inflammation model)	10, 25, 50, 100 μg/mL	10 kDa	Rha, GalA, Glu, Gal, Ara 0.26∶0.14∶24.04∶0.62∶1		NO ↓, TNF-α ↓, IL-10 ↑	Closely related to amino acid metabolism and energy metabolism	[127]
*Astragalus membranaceu*	APS-1	C57BL/6 mice (T1D model)	200 mg/kg 8 consecutive weeks				IL-10 ↑, IL-6 ↓, TNF-α ↓, SCFAs ↑, BCFAs ↓, GPR41 ↑, HDAC2 ↑, ZO-1 ↑, occludin ↑, claudin-1 ↑	Alleviates T1D system inflammation by reducing inflammatory factors and regulating gut microbes	[123]
*Astragalus membranaceu*	APS-A1	RAW264.7 cell (LPS-induced inflammation model)	50, 100, 200 μg/mL dependent manner	2620 KDa	Glu, Gal, Ara 52.3:1.0:1.3.	1,4-α-D-Glcp	TNF-α ↓, IL-1β ↓, IL-6 ↓, MCP-1 ↓, NLRP3 ↓, iNOS ↓, COX-2 ↓, p-JNK ↓, p-ERK ↓, p-p38 ↓, P65 ↓	Alleviates LPS-induced inflammation by inhibiting the MAPK and NF-κB signaling pathways	[128]
*Astragalus membranaceu*	APS-B1	RAW264.7 cell (LPS-induced inflammation model)	50, 100, 200 μg/mL Dependent manner	4950 KDa.	Glu, Gal, Ara, Man, Rha, GalA 75.2:17.3:19.4:1.0:1.1:1.3	1,4-α-D-Glcp,1,4,6-α-D-Glcp,1,5-α-L-Araf	TNF-α ↓, IL-1β ↓, IL-6 ↓, MCP-1 ↓, NLRP3 ↓, iNOS, ↓COX-2 ↓, p-JNK ↓, p-ERK ↓, p-p38 ↓, P65 ↓	Alleviates LPS-induced inflammation by inhibiting the MAPK and NF-κB signaling pathways	[128]
*Astragalus membranaceu*	AP	C57BL/6 mice (CVB3-induced viral myocarditis model)	200 mg/kg				IL-1β ↓, IL-6 ↓, TNF-α ↓, INF-γ ↓, MCP-1 ↓, TLR-4 ↓, p-NF-κB p65 ↓	Alleviation of CVB3-induced viral myocarditis by inhibiting the TLR-4/NF-κB p65 signaling pathway	[20]
*Astragalu membranaceu*	APSI-C	RAW264.7 cell (LPS-induced inflammation model)	12.5, 25, 50 mg/L	4.5 KDa			TNF-α ↓, NO ↓, IL-10 ↑	Alleviating LPS-induced inflammation by inhibiting inflammatory factors and increasing levels of pro-inflammatory factors	[129]
*Astragalus membranaceu*	sAPS3	Wistar rats (CCl4-induced hepatocellular necrosis model)	40 mg/kg 3 weeks				TNF-α ↓, IL-β1 ↓, ATG7 ↓, CD68 ↓, LC3II ↓	Alleviating CCl4-induced liver injury by inhibiting inflammatory factors and decreasing the expression levels of ATG7 or LC3II, key regulators of Kupffer (KCs) autophagy	[130]
*Astragalus membranaceu*	SAPS	Caco2 cell (LPS-induced inflammation model)	25, 50, 100 μg/mL				TLR4 ↓, TNF-α ↓, IL-1β ↓, IL-8 ↓, ZO-1 ↑, Occludin ↑	Alleviating LPS-induced inflammation by inhibiting inflammatory factors and modulating intestinal flora	[131]
*Ganoderma lucidum*	BSGLP	C57BL/6 J mice (HFD-induced obesity model)	100, 300 mg/kg	26.0 kDa	Glu, Man, Gal 87.4:4.81:8.14	(1→3)-β-D-Glcp, (1→6)-β-D-Glcp, (1→3,6)-β-D-Glcp	IL-1β ↓, IL-6 ↓, MCP-1 ↓, Occludin ↑, ZO-1 ↑, Claudin-1 ↑, SCFAs ↑, LBP ↓, CD14 ↓, Myd88 ↓, TLR4 ↓, p-NF-κB ↓, GPR43 ↑, Firmicutes/Bacteroidetes ↓, Reg3γ ↓	Alleviation of inflammation through the modulation of gut microbes and inhibition of the TLR4/Myd88/NF-κB signaling pathway	[36]
*Ganoderma lucidum*	GLP-1	Wistar rats (D-gal induced cognitive impairment model)	20 mg/kg 10 mL/kg 60 days	107 KDa		(1→, and →3)-β-D-Glcp	p-p38MAPK ↓, p-p53 ↓, p-JNK1+JNK2+JNK3 ↓, TNF-α ↓, IL-6 ↓, IL-10 ↑, TGF-β1 ↑	Alleviating D-gal-induced systemic inflammation by inhibiting the MAPK signaling pathway and reducing inflammatory factors	[55]
*Ganoderma lucidum*	SGRP	Kunming mice (CCl4-induced chronic liver injury model)	400, 200, 100 mg/kg 6 weeks	15.542 KDa	Fuc, Xyl, Man, Gal, Glu 4.8:0.9:4.9:9.9:11.6	(1 → 6)-linked glycoside	TNF-α ↓, IL-1β ↓, IL-6 ↓, TLR4 ↓, p-NF-κB p65 ↓, IκBα ↑	Alleviation of liver fibrosis by inhibiting the TLR4/NF-κB signaling pathway	[132]
*Ganoderma lucidum*	GRP	Kunming mice (CCl4-induced chronic liver injury model)	400, 200, 100 mg/kg 4 weeks	12.2 kDa	Rha, Fuc, Man, Glu 1.99:1.21:6.33:6.78		TNF-α ↓, IL-6 ↓, IL-10 ↓, p-p65 ↓, TGF-β ↓, IκBα ↑	Alleviating chronic liver injury by reducing pro- and anti-inflammatory factors	[133]
*Ganoderma lucidum*	PSG-1	BALB/c mice (cyclophosphamide-induced intestinal mucosal dysfunction model)	25, 50, 100 mg/kg 7 days				TLR-2 ↓, TLR-4 ↓, TLR-6 ↓, IFN-γ ↑, IL-2 ↑, IL-12p70 ↑, IL-4 ↑, IL-1β ↑, IL-17 ↑, IL-21 ↑, IL-23 ↑, TGF-β3 ↑, T-bet ↑, GATA-3 ↑, RORγt ↑, Foxp3 ↑, ZO-1 ↑, occludin ↑, claudin-1 ↑, LC3 ↑, Beclin-1 ↑, Atg5 ↑, Atg7 ↑	Alleviating cyclophosphamide (Cy)-induced intestinal mucosal dysfunction by regulating intestinal flora and improving intestinal immunity	[39]
*Ganoderma lucidum*	GLP	C57BL/6 mice (AOM/DSS-induced inflammation, tumorigenesis model) RAW264.7, HT-29, NCM460 cell (LPS-induced inflammation model)	200, 300 mg/kg 0.8 mg/mL	25.0 kDa	Ara, Man, Glu, Gal (4.19%), (15.69%), (78.15%), (1.97%)		TLR4 ↓, p-NF-κB p65 ↓, Myd88 ↓, IL-1β ↓, iNOS ↓, COX-2 ↓, p-JNK ↓, p-ERK ↓, IL-6 ↓, IL-1β5, TNF-α ↓, SCFAs ↑, occludin ↑, ZO-1 ↑	Regulation of intestinal flora through inhibition of MAPK and NF-κB and increased production of SCFAs to alleviate colitis and tumors	[61]
*Ganoderma lucidum*	GLP	C57BL/6 mice (CPZ-induced CNS demyelinating disease model) (MOG35-55 induces the development of an experimental autoimmune encephalomyelitis disease model) BV2cell (LPS-induced neuroinflammation model)	5 mg/kg 50 μg/mL				NF-κB ↓, NLRP3 ↓, ASC ↓, pro-caspase-1 ↓, caspase-1 ↓, IL-1β ↓, TNFα ↓, IL-17 ↓, Dectin-1 ↑, IL-10 ↑	Regulation of the Dectin-1 receptor inhibits NF-κB/NLRP3 inflammatory vesicle signaling and thus suppresses neuroinflammation	[75]
*Ganoderma lucidum*	CM-GLP	SD rats (cerebral ischemia-reperfusion model)	40 mg/kg				MDA ↓, NF-κB ↓, TNF-α ↓, IL-1 ↓, IL-6 ↓, SOD ↑, HSP-70 ↑, p-Akt ↑	Alleviating cerebral ischemia-reperfusion injury by modulating the HSP70/PI3K/Akt signaling pathway	[134]
*Ganoderma lucidum*	GLPN	C57 mice (DSS-induced colitis model)	200 mg/kg 17 days	35 KDa	Glc	(1→3)-β-D- glucan, (1→6)-β-D- l side-branching unit on every third residue	TNF-α ↓, IL-1β ↓, IL-6 ↓	Relief of colitis by inhibiting L-selectin binding to ligands	[135]
*Dendrobium nobile*	DNP1	RAW264.7 cells (LPS-induced inflammation model)	200 μg/mL	67.72 kDa	Man, Glc (75.86%), (24.14%)	β-1,4-ᴅ-Manp, β-1,4-ᴅ-Glcp residues	NO ↓, TNF-α ↓, IL-1β ↓, IL-6 ↓, IL-10 ↑	Alleviating LPS-induced inflammation by modulating pro- and anti-inflammatory factors	[136]
*Dendrobium nobile*	DNP2	RAW264.7 cells (LPS-induced inflammation model)	200 μg/mL	37.45 kDa	Man, Glc (72.32%), (27.68%)	β-1,4-ᴅ-Manp, β-1,4-ᴅ-Glcp residues	NO ↓, TNF-α ↓, IL-1β ↓, IL-6 ↓, IL-10 ↑	Alleviating LPS-induced inflammation by modulating pro- and anti-inflammatory factors	[136]
*Dendrobium huoshanense*	cDHPS	DBA/1J male mice (type II collagen-induced arthritis model)	0.1095, 0.4380 g/kg 30 days dose-dependent manner				p-IκB ↓, p- p65 ↓, p-JNK ↓, p-p38 ↓, p-ERK1/2 ↓, p-PI3K ↓, p- AKT ↓, p-JAK1 ↓, p- STAT3 ↓, IL-1β ↓, IL-6 ↓, IL-17 ↓, TNF-α ↓, GM-CSF ↓, M-CSF ↓, CXCL12 ↓, CCL ↓5, MMP3 ↓, MMP8 ↓, MMP9 ↓, VEGF ↓, IL-10 ↑, TGF-β ↑, HIF-1α ↓	Alleviation of rheumatoid arthritis through inhibition of the NF-κB, MAPK, PI3K/AKT, and JAK1/STAT3 signaling pathways	[22]
*Dendrobium officinale*	DOPS	BalB/c mice (4% DSS-induced secondary liver injury in an acute colitis model) RAW264.7 cells (LPS-induced inflammation model)	50, 100, 200 mg/kg 14 days 50, 100, 200 μg/mL	393.8 kDa	Man, Glu 5.83:1.05		IL-1β ↓, TNF-α ↓, MDA ↓, SOD ↑, GSH-Px ↑, Nrf-2 ↑, HO-1 ↑, NQO-1 ↑	Alleviation of liver injury secondary to colitis by activation of the Nrf-2 signaling pathway	[101]
*Dendrobium officinale*	DOPS	Kunming mice (ovariectomy, D-gal-induced learning and memory impairment model)	140 mg/kg				MDA ↓, TNF-α ↓, IL-1β ↓, Nrf2 ↑, HO-1 ↑	Improving Learning Memory Disorders by Activating the Nrf2/HO-1 Signaling Pathway	[102]
*Dendrobium officinale*	M-DOP	Kunming mice (D-Gal-induced aging model)	250, 500, 1000 mg/kg	75.41 kDa	Ara, Gal, Glc, Man, Rha 0.38:0.40:1.00:0.12:0.02		SOD ↑, CAT ↑, GSH-Px ↑, Nrf2 ↑, HO-1 ↑, NQO1 ↑, IL-6 ↓, IL-1β ↓, NO ↓	Amelioration of liver injury by activation of Nrf2/HO-1/NQO1 signaling pathway	[100]
*Dendrobium officinale*	DOP	SD rats (middle cerebral artery occlusion model)	25, 50, 100 μg/g				IFN-γ ↓, COX-2 ↓, IL-6 ↓, p-JAK/JAK ↓, p-STAT3/STAT3 ↓	Reduces brain inflammation and repairs neurological function by inhibiting JAK/STAT3 signaling pathway activation	[113]
*Dendrobium officinale*	DOP	BALB/c mice (DSS-induced colitis model) Caco-2, RAW264.7 cells (LPS-induced inflammation model)	200 mg/kg 20 days 0.5 mg/mL	618 kDa		1,4-β-D-mannopyranosyl residues, β-D glucopyranosyl residue	miR-433-3p ↑, NO ↓, TNF-α ↓, IL-6 ↓, PGE2 ↓, MAPK8 ↓	Alleviating intestinal inflammation by inhibiting the MAPK signaling pathway	[125]
*Dendrobium huoshanense*	DHP-1	RAW264.7 cells (LPS-induced inflammation model)	25, 50, 100, 200, 400 μg/mL	262.50 kDa	Gal, Man, Glc, 1.00:1.89:22.66		NO ↓, IL-1β ↓	Alleviating LPS-induced inflammation by inhibiting pro-inflammatory factors	[137]
*Dendrobium huoshanense*	DHP-2	RAW264.7 cells (LPS-induced inflammation model)	25, 50, 100, 200, 400 μg/mL	521.37 kDa	Gal, Man, Glc, 2.80:1.00:10.93		NO ↓, IL-1β ↓	Alleviating LPS-induced inflammation by inhibiting pro-inflammatory factors	[137]
*Lycium chinense*	LBPs	SD rats (nonalcoholic fatty liver disease model)	50 mg/kg 8 weeks		Man, Rha, Glu, Gal, Ara 1.00:0.93:12.55:0.31:0.53		IL-6 ↓, TNF-α ↓, IL-1β ↓, MCP-1 ↓, IL-10 ↑, TLR4 ↓, MyD88 ↓, IKK ↓, IκB ↓, p38MAPK ↓, NF-κBp65 ↓, occludin ↑, ZO-1 ↑	Alleviation of NAFLD by inhibition of TLR4/MyD88/NF-κB and MAPK and modulation of intestinal flora	[23]
*Lycium chinense*	LBPs	SD rats (nonalcoholic fatty liver disease model)	1 mg/kg 8 weeks				iNOS ↓, COX-2 ↓, IL-1β ↓, SOCS-3 ↓, TGF-β1 ↓, a-SMA ↓, p-JNK ↓, p-c-Jun ↓, p-ERK ↓, p-MEK ↓	Alleviating NAFLD by inhibiting the MAPK signaling pathway	[62]
*Lycium chinense*	LBP	Bovine mammary epithelial cells (LPS-induced inflammation model)	100, 300 μg/mL 24 h				COX-2 ↓, NLRP3 ↓, TNF-α ↓, IL-1β, IL-6 ↓, IκBα ↓, p65 ↓, p38 ↓, JNK ↓, ERK ↓, PPARγ ↑	Mitigation of mastitis by inhibiting the MAPK/NF-κB signaling pathway in a PPARγ-dependent manner	[90]
*Lycium chinense*	GDLP	C57BL/KsJ mice (T2DM model)	400 mg/kg 8 weeks				TNF-α ↓, Nrf2 ↓, HO-1 ↓	Alleviating type 2 diabetes-induced liver inflammation by inhibiting the Nrf2/HO-1 signaling pathway	[105]
*Lycium chinense*	LBPs	RAW264.7 cell (LPS-induced inflammation model)	1 g/L 24 h	34.6 KDa			NO ↓	Alleviating LPS-induced inflammation by inhibiting NO secretion levels	[138]
*Angelica sinensis*	AP	HT22 cell (LPS-induced inflammation model)	80μg/mL				IL-1β ↓, TNF-α ↓, IL-6 ↓, miR-10a ↑, p-IκBa ↓, p-p65 ↓, pJAK2 ↓, p-STAT3 ↓, p53 ↓, p21 ↓, cleaved PARP ↓, cleaved caspase-3/9 ↓	Alleviating LPS-induced inflammatory injury by inhibiting the NF-κB and JAK2/STAT3 signaling pathways and modulating miR-10a	[111]
*Angelica sinensis*	APS-2I	BalB/c mice (septicemia model) RAW264.7 cell (LPS-induced inflammation model)	5, 10 mg/L 20, 40 mg/L	720 KDa	Man, Rha, Glc, Gal, Ara, GalA (4.9%), (6.5%), (1.2%), (12.2%), (28.0%), (47.2%)	α-1,5-Araf, α-1,3-Araf, α-1,3,5-Araf, β-1,4-Galp, β-1,6-Galp	TNF-α ↓, IFN-β ↓, NO ↓, TIRAP ↓, MyD88 ↓, TRAM ↓, TRIF ↓, TLR4 ↓, MD-2 ↓	Relief of sepsis by inhibition of the TLR4/Myd88/NF-κB signaling pathway and TRAM/TRIF signaling pathway	[139]
*Angelica sinensis*	APS-3I	BalB/c mice (septicemia model) RAW264.7 cell (LPS-induced inflammation model)	5, 10 mg/L 20, 40 mg/L	590 KDa	Mainly Glc	α-1,6-Glcp, α-1,2-Glcp, α-1,3-Glcp	TNF-α ↓, IFN-β ↓, NO ↓, TIRAP ↓, MyD88 ↓, TRAM ↓, TRIF ↓, TLR4 ↓, MD-2 ↓	Relief of sepsis by inhibition of the TLR4/Myd88/NF-κB signaling pathway and TRAM/TRIF signaling pathway	[139]
*Angelica sinensis*	AP	Primary claw dermal cells (LPS-induced inflammation model)	10, 50, 100 µg/mL				p-IκBα ↓, p-p65 ↓, p-ERK ↓, p-JNK ↓, p-p38 ↓, CCL2 ↓, CCL20 ↓, CXCL2 ↓, CXCL8 ↓, CXCL10 ↓, TLR4 ↓, MyD88 ↓, TNF-α ↓, IL-1β ↓, IL-6 ↓, NO ↓	Alleviating LPS-induced inflammation by inhibiting the NF-κB and MAPK signaling pathways	[56]
*Angelica sinensis*	AP	SD rats (chronic renal failure model)	10, 20, 40 mg/mL dose-dependent manner				IL-18 ↓, IL-1β ↓, IL-6 ↓, NLRP3 ↓, caspase-1 ↓	Alleviating chronic functional renal failure by inhibiting NLRP3 inflammasome signaling activation	[77]
*Angelica sinensis*	sCAP	ICR mice (CCl4-induced hepatic injury model)	0.05, 0.1, 0.15 mg/mL				p-ERK ↓, p-JNK ↓, p-p38 ↓, MDA ↓, ROS ↓, SOD ↑, T-AOC ↑	Mitigation of CCl4-induced liver injury by MAPK inhibition	[140]
*Angelica sinensis*	CAP	ICR mice (CCl4-induced hepatic injury model)	0.05, 0.1, 0.15 mg/mL				p-ERK ↓, p-JNK ↓, p-p38 ↓, MDA ↓, ROS ↓, SOD ↑, T-AOC ↑	Mitigation of CCl4-induced liver injury by MAPK inhibition	[140]
*Polygonatum sibiricum*	PSP	BALB/c mice (septic acute liver injury model)	150, 300, 600 mg/kg				TNF-α ↓, IL-6 ↓, MPO ↓, IL-18 ↓, IL-1β ↓, NLRP3 ↓, ASC ↓, caspase-1 ↓, AST ↓, ALT ↓, ALP ↓, TBIL ↓	Treatment of septic acute liver injury by inhibiting the NLRP3/GSDMD signaling pathway	[76]
*Polygonatum sibiricum*	PCP	KM mice (LPS-induced acute lung injury model)	400, 800 mg/kg/dw 7 consecutive days	8.842 KDa	Fru, Glu, Gal 92.73:6.37:0.90	β-D, α-D	IL-1β, IL-6, TNF-α, MPO ↓, SOD ↑, p-IKKβ ↓, p-IκBα ↓, p-p65 ↓, HO-1 ↓, NQO-1 ↓, Nrf2 ↓, p-AMPK ↓	Lung protection through inhibition of the NF-κB and AMPK-Nrf2 signaling pathways	[141]
*Polygonatum sibiricum*	HPCP	KM mice (LPS-induced acute lung injury model)	400, 800 mg/kg/dw 7 consecutive days	5.521 KDa	Fru, Glu, Gal, Ara, Xyl 60.16:22.35:13.03:1.35:3.12	β-D, α-D	IL-1β, IL-6, TNF-α, MPO ↓, SOD ↑, p-IKKβ ↓, p-IκBα ↓, p-p65 ↓, HO-1 ↓, NQO-1 ↓, Nrf2 ↓, p-AMPK ↓	Lung protection through inhibition of the NF-κB and AMPK-Nrf2 signaling pathways	[141]
*Polygonatum sibiricum*	PCP	SD rats (CCl4-induced acute liver injury model)	400 mg/kg 7 consecutive days				GSH ↑, SOD ↑, ROS ↓, MDA ↓, p-PI3K/PI3K ↓, p-AKT/AKT ↓, p-m TOR/mTOR ↓, LC3II/LC3I ↑	Attenuating CCl4-induced acute liver injury by activating autophagy through inhibition of the PI3K/AKT/mTOR pathway	[87]
*Polygonatum sibiricum*	PSP	C57BL/6 mice (Single prolonged stress model)	200, 400, 800 mg/kg	6–14 kD			IL-1β ↓, TNF-α ↓, NLRP3 ↓, ASC ↓, SOD ↑, MDA ↓, HO-1 ↓, Nrf2 ↓, BDNF ↑, p-TrkB ↑, PSD95 ↑, Arc ↑, GluA1 ↑, GluN2B ↓	Attenuating PTSD-like behaviors by inhibiting activation of Nrf2/HO-1, inhibiting the NLRP3 signaling pathway	[104]
*Polygonatum sibiricum*	PSP	C57BL/6 mice (LPS and chronic unpredictable mild stress-induced depression model)	100, 200, 400 mg/kg	6–14 kD	Ara, Glu, GluA, Gal, GalA, Man, Rha, Rib 13.7:82.9:3.7:36.2:4.3:52.5:3.3:1.0		GluA1 ↑, GluA2 ↑, GluN2A ↓, GluN2B ↓, p-AKT/AKT ↑, p-mTOR/mTOR ↑, caspase-3 ↓, IL-1β ↓, TNF-α ↓, p-ERK ↓, NF-κB ↓, SOD ↑, MDA ↓, CORT ↓, 5-HT ↑	Prevent depression by reducing inflammation by inhibiting the NF-κB and MAPK signaling pathways	[142]
*Polygonatum sibiricum*	PS	SD rat (HFD-induced obesity model)	120, 240, 480 mg/kg 14 weeks	134.7 kDa	Man, Rha, GalA, Gal, Glc, GlcA, Xyl, Ara, Fuc, idoA		ZO-1 ↑, occludin ↑, TLR4 ↓, IL-1β ↓, IL-10 ↑, IκB-α ↑, SCFA ↑	Alleviating inflammation by inhibiting TLR4/NFκB and modulating intestinal flora	[126]
*Polygonatum sibiricum*	PSF	SD rat (HFD-induced obesity model)	120, 240, 480 mg/kg 14 weeks	178.6 kD	Man, Rha, GalA, Gal, Glc, GlcA, Xyl, Ara, Fuc, idoA		ZO-1 ↑, occludin ↑, TLR4 ↓, IL-1β ↓, IL-10 ↑, IκB-α ↑, SCFA ↑	Alleviating inflammation by inhibiting TLR4/NFκB and modulating intestinal flora	[126]
*Phellinus igniarius*	S-A3	C57BL/6 mice (ulcerative colitis model) RAW264.7 (LPS-induced inflammation model)	50, 100 mg/kg 31.25, 15.625, 7.8125 μg/mL	3.3 KDa	Gal, Glc, Man, GlcA contain small amounts of Fuc, Xyl, GalA, Rha		TNF-α ↓, IL-6 ↓, IL-1β ↓, p65 ↓, AKT ↓, JNK ↓, P38 ↓	Inhibit ulcerative colitis by inhibiting the NF-κB, MAPK, and AKT signaling pathways	[143]
*Phellinus igniarius*	SHPS-1	C57BL/6 mice (ulcerative colitis model) RAW264.7 cells (LPS-induced inflammation model)	100 mg/kg 28 day 250 μg/mL 24 h	46 kDa	Ara, Man, Glu, Gal 2.2:15.7:49.3:32.8	1,3-linked β-D-Glcp 1,6-linked α-D-Galp residues	IL-1β ↓, TNF-α ↓, IL-10 ↑, iNOS ↓, INF-β ↓, INF-γ ↓, MCP-1 ↓, CXCL-1 ↓, CD 86 ↓, IL-4 ↑, Occludin ↑, Claudin-4 ↑, ZO-1 ↑, CD 206 ↑, p-STAT-1 ↓	Ulcerative colitis is inhibited by reducing the phosphorylation level of STAT-1 and the expression level of STAT-1 target genes such as iNOS and TNF-α, as well as increasing the anti-inflammatory factor and CD206	[144]
*Phellinus igniarius*	PLP	ICR mice (enteritis model) RAW264.7 cells (LPS-induced inflammation model)	500 mg/kg 25, 50, 100 μg/mL				NO ↓, MPO ↓, MDA ↓, IL-1β ↓, TNF-α ↓, iNOS ↓, IL-6 ↓, p38 ↓, JNK ↓, ERK ↓, PPARα ↑, PPARγ ↑	By activating PPARα and PPARγ, MAPK signaling pathway is blocked to alleviate inflammation	[93]
*Phellinus igniarius*	SHP-1-1	RAW264.7 cells (LPS-induced inflammation model)	25, 50, 100 μg/mL	333.599 kDa	Fuc, Ara, Rha, Gal, Glu, Xyl, Man, Glu 11.48∶0.18∶0.28∶17.86∶27.71∶0.64∶11.75∶0.94	α-Glycosidic bond	NO ↓, TNF-α ↓, IL-6 ↓, IL-1β ↓	Reduces inflammation by inhibiting pro-inflammatory factors	[145]
*Phellinus igniarius*	SHP-2-1	RAW264.7 cells (LPS-induced inflammation model)	25, 50, 100 μg/mL	563.032 kDa	Fuc, Ara, Rha, Gal, Glu, Xyl, Man, Glu 0.73∶0.14∶0.32∶1.13:14.96∶1.04∶3.79∶1.50	β-Glycosidic bond	NO ↓, TNF-α ↓, IL-6 ↓, IL-1β ↓	Reduces inflammation by inhibiting pro-inflammatory factors	[145]
*Poria cocos*	PPS	KM mice BV-2 cell (LPS-induced anxiety and depression-like behavior model)	20, 80 mg/kg 4, 8, 16 μmol/L				ROS ↓, NO ↓, TNF-α ↓, IL-1β ↓, CD16/32 ↓, NF-κB p65 ↓, CD206 ↑, NLRP3 ↓, ASC ↓, cleaved caspase-1 ↓	Attenuating LPS-induced anxiety and depression-like behaviors by inhibiting NF-κB and NLRP3 signaling pathways.	[74]
*Poria cocos*	PCP	Sheep renal tubular epithelial cells (MAP-induced inflammation and oxidative stress model)	5, 10, 50 mg/L				MDA ↓, SOD ↑, IL-6 ↓, TNF-α ↓, Nrf2 ↑, HO-1 ↑, NQO1 ↑	Reduces inflammation by activating the Nrf2/HO-1 signaling pathway	[103]
*Poria cocos*	PCP-1C	KM mice (CCl4-induced liver injury model)	50, 100, 200 mg/kg Two consecutive weeks	17 kDa	Man, Gal, Glc, Fuc 17.4, 43.5, 24.4, 14.6	1,3-β-D-Glcp, 1,4-β-D-Glcp, 1,6-β-D-Glcp,	IL-1β ↓, IL-6 ↓, TNF-α ↓, SOD ↑, GSH-Px ↑, MDA ↓, CAR ↓, CYP2E1 ↓	Alleviation of CCl4-induced liver injury by inhibiting CAR/CYP2E1 signaling pathway	[146]
*Poria cocos*	CMP44	RAW264.7 cell (LPS-induced inflammation model)	31.25–1000 μg/mL	209.6 KDa	D-glucose	β- (1, 3)	NO ↓, TNF-α ↓, IL-6 ↓, IL-1β ↓	Reduces inflammation by inhibiting pro-inflammatory factors	[147]
*Poria cocos*	CMP33	RAW264.7 cell (LPS-induced inflammation model)	31.25–1000 μg/mL	152.3 KDa		(1 → 3), (1→6), (1→2)-linked glucose residues	NO ↓, TNF-α ↓, IL-6 ↓, IL-1β ↓	Reduces inflammation by inhibiting pro-inflammatory factors	[148]

**Table 3:** Molecular weight of MEHTCMPs with anti-inflammatory effect.

Source	Compound Name	Molecular Weight	Effects	References
*Lycium barbarum*	LBP	34.6 KDa	NO ↓	[138]
*Angelica sinensis*	ASP-Hb	67.9 KDa	IL-6 ↓, IL-1β ↓, TNF-α ↓, TLR4 ↓	[160]
honey of *Polygonatum sibiricum Delar. ex Redoute*	HPCP	5521 KDa	p-IKKβ ↓, p-IκBα ↓, p-p65 ↓, IL-1β ↓, TNF-α ↓, IL-6 ↓, p-AMPK ↑, Nrf2 ↑, HO-1 ↑, NQO-1 ↑	[141]
*Astragalus* *membranaceus*	APSI-C	4.5 KDa	NO ↓, TNF-α ↓	[129]
*Dendrobium huoshanense*	DHP-1	521.37 KDa	NO ↓, IL-1β ↓	[137]
*Dendrobium huoshanense*	DHP-2	262.50 KDa	NO ↓, IL-1β ↓	[137]

**Table 4:** Composition and Proportion of Monosaccharides of MEHTCMPs with anti-inflammatory effect.

Source	Compound Name	Composition and Proportion of Monosaccharides	Effects	References
*Dioscorea polystachya*	CYP-1	Rib, Rha, Ara, Xyl	TNF-α ↓, IL-1β ↓	[163]
*Rubusidaeus*	L-Ps-1	Rha, Ara, Xyl, glucose, galactose 2.47:4.75:4.12:1:2.48	TNF-α ↓, iNOS ↓, IL-6 ↓	[165]
*Rubusidaeus*	F-Ps-3	Rha, Ara, Xyl, Glu, Gal 4.21:14.72:1.63:1:3.22	TNF-α ↓, iNOS ↓, IL-6 ↓	[165]
*Astragalus membranaceus*	APS-I	Man, Rha, Gal A, Glu, Gal, Ara 0.54:0.26:12.24:17.24:8.46:1	NO ↓, TNF-α ↓, IL-10 ↑	[127]
*Astragalus membranaceus*	APS-II	Rha, Gal A, Glu, Gal, Ara 0.26:0.14:24.04:0.62:1	NO ↓, TNF-α ↓, IL-10 ↑	[127]
*Phellinus igniarius*	SHP-2-1	Fuc, Ara, Rha, Gal, Glucose, Xyl, Man, Glu A 0.73:0.14:0.32:1.13:14.96:1.04:3.79:1.50	NO ↓, IL-1β ↓	[145]
*Phellinus igniarius*	SHP-1-1	Fuc, Ara, Rha, Gal, Glu, Xyl, Man, Glu A 11.48:0.18:0.28:17.86:27.71:0.64:11.75:0.94	NO ↓, IL-1β ↓	[145]
*Sargassum pallidum*	PPS	Fucose	NO ↓	[166]
*Dendrobium nobile*	DNP1	Man (75.86%), Glc (24.14%)	NO ↓, TNF-α ↓, IL-1β ↓, IL-6 ↓, IL-10 ↑	[136]
*Dendrobium nobile*	DNP2	Man (72.32%), Glc (27.68%)	NO ↓, TNF-α ↓, IL-1β ↓, IL-6 ↓, IL-10 ↑	[136]

**Table 5:** Glucosidic Bond of MEHTCMPs with anti-inflammatory effect.

Source	Compound Name	Glucosidic Bond	Effects	References
*Pueraria montana var. thomsonii*	RPP-2	α-D-1,3-glucan	TNF-α ↓	[170]
*Hericium erinaceus*	EP-1	β-d-Glc(1→3)	SOD ↑, ROS ↓	[171,172]
*Phellinus igniarius*	SHPS-1	1, 3-β-D-GLCP residue	STAT-1 ↓, iNOS ↓, TNF-α ↓	[144]
*Phellinus igniarius*	A3	α-1, 6-D-GALp	IL-6 ↓, IL-1β ↓, TNF-α ↓, P65 ↓, p-P38 ↓, p-ERK ↓, p-JNK ↓, p-AKT ↓	[143]
honey	AHPN50-1a	(1→6) -α-GlcP	IL-1β ↓, IL-6 ↓, TNF-α ↓	[173]
*Poria cocos*	PCP-1C	1,3-β-D-Glcp	IL-1β ↓, IL-6 ↓, TNF-α ↓, SOD ↑, GSH-Px ↑	[146]
*Ganoderma lucidum*	MBG	β-1→3 and β-1→6 glucan	IgA ↑, IgG ↑, poly-Ig ↑, IL-2 ↑	[174]
*Angelica sinensis*	APS-2I	α-D-β-Galp-(1→6)	MyD88 ↓, TLR4 ↓, TNF-α ↓, IFN-β ↓, IL-6 ↓, NO ↓	[139]
*Poria cocos*	CMP44	(1→3) -β-d-glucan, (1→6)-β,(1→2)-β glucoside bonds	NO ↓, TNF-α ↓, IL-6 ↓, IL-1β ↓	[147]
*Ganoderma lucidum*	BSGLP	(1→3)-β-D-Glcp, (1→6)-β-D-Glcp	TLR4 ↓, Myd88 ↓, NF-κB ↓	[36]

**Table 6:** Advanced Structure of MEHTCMPs with anti-inflammatory effect.

Source	Compound Name	Conformation	Appearance Characteristics	Effects	References
*Poria cocos*	CMP33	triple helix structure		IL-6 ↓, TNF-α ↓, IL-1β	[148]
*Ganoderma lucidum*	GLP	triple helix structure		TNF-α ↓, IL-1β ↓, IL-6 ↓, L-selectin ↓	[135]
*Pueraria montana var. thomsonii*	RPP-2		smooth, clean, and irregular sheet structure	TNF-α ↓	[170]
*Gardenia jasminoides*	GPS		irregular, thin, randomly distributed, and amorphous structures	TLR4 ↓, NF-κB ↓, MyD88 ↓, MCP-1 ↓, IL-6 ↓	[177]
*Pseudocydonia sinensis*	CSP-M		a sheet surface and porous structures	MPO ↓, TNF-α ↓, IL-1β ↓, IL-6 ↓, NO ↓, MDA ↓, SOD ↑, GSH ↑	[178]
*ginger*	GP-Zn(II)		flat surface, sheet structure, and partial dendritic fragments	IL-1β ↓, IL-6 ↓, IL-8 ↓, IL-12 ↓, TNF-α ↓, IL-10 ↑	[179]

## Data Availability

Data are contained within the article.

## Abbreviations

Glc	Glucose
Gal	Galactose
Rha	Rhamnose
Ara	Arabinose
Man	Mannose
GalA	Galacturonic acid
Xyl	Xylose
Rib	Ribose
GlcA	Glucuronic acid
Fuc	Fucuronic
Fru	Fructose
Idoa	Iduronic acid
NF-κB	Nuclear factor-κB
NLRP3	Nucleotide-binding oligomerization domain leucine-rich repeat and pyrin domain-containing 3
TNF-α	Tumor necrosis factor-α
Caspase-1	Cysteinyl aspartate specific proteinase-1
IL-1β	Interleukin
ERK	Extracellular signal-regulated protein kinase
JNK	c-Jun N-terminal kinase
MAPK	Mitogen-activated protein kinase
Bcl2	B-cell lymphoma-2
Bax	bcl2-Associated X
Nrf2	Nuclear factor erythroid 2-related factor 2
NQO-1	NAD(P)H quinone dehydrogenase-1
HO-1	Heme oxygenase-1
SOD	Superoxide dismutase
Interleukin	(IL)-1β/4/6/10/12/B
AP-1	Activator protein-1
PYY	Peptide YY
SCFAs	Short chain fatty acids
ZO-1	Zona occludens 1
GPR41/43	G-protein-coupled receptor 41/43
TLR4	Toll-like receptor 4
LPS	Lipopolysaccharide
PPARs/PPARα/PPARγ	Peroxisome proliferators-activated receptor-s/α/γ
PI3K	Phosphoinositide-3 kinase
Akt	Protein kinase B
GSK3β	Glycogen synthase kinase
mTOR	Mammalian target of rapamycin
JAK	Janus activated kinase
STAT	Signal transducer and activator of transcription
